# miR-140-5p in Small Extracellular Vesicles From Human Papilla Cells Stimulates Hair Growth by Promoting Proliferation of Outer Root Sheath and Hair Matrix Cells

**DOI:** 10.3389/fcell.2020.593638

**Published:** 2020-12-14

**Authors:** Yuxin Chen, Junfei Huang, Zhen Liu, Ruosi Chen, Danlan Fu, Lunan Yang, Jin Wang, Lijuan Du, Lihong Wen, Yong Miao, Zhiqi Hu

**Affiliations:** Department of Plastic and Aesthetic Surgery, Nanfang Hospital of Southern Medical University, Guangzhou, China

**Keywords:** extracellular vesicles, hair growth, dermal papilla cells, microRNA, BMP signaling

## Abstract

The application of dermal papilla cells to hair follicle (HF) regeneration has attracted a great deal of attention. However, cultured dermal papilla cells (DPCs) tend to lose their capacity to induce hair growth during passage, restricting their usefulness. Accumulating evidence indicates that DPCs regulate HF growth mainly through their unique paracrine properties, raising the possibility of therapies based on extracellular vesicles (EVs). In this study, we explored the effects of EVs from high- and low-passage human scalp follicle dermal papilla cells (DP-EVs) on activation of hair growth, and investigated the underlying mechanism. DP-EVs were isolated by ultracentrifugation and cultured with human scalp follicles, hair matrix cells (MxCs), and outer root sheath cells (ORSCs), and we found low-passage DP-EVs accelerated HF elongation and cell proliferation activation. High-throughput miRNA sequencing and bioinformatics analysis identified 100 miRNAs that were differentially expressed between low- (P3) and high- (P8) passage DP-EVs. GO and KEGG pathway analysis of 1803 overlapping target genes revealed significant enrichment in the BMP/TGF-β signaling pathways. BMP2 was identified as a hub of the overlapping genes. miR-140-5p, which was highly enriched in low-passage DP-EVs, was identified as a potential regulator of BMP2. Direct repression of BMP2 by miR-140-5p was confirmed by dual-luciferase reporter assay. Moreover, overexpression and inhibition of miR-140-5p in DP-EVs suppressed and increased expression of BMP signaling components, respectively, indicating that this miRNA plays a critical role in hair growth and cell proliferation. DP-EVs transport miR-140-5p from DPCs to epithelial cells, where it downregulates BMP2. Therefore, DPC-derived vesicular miR-140-5p represents a therapeutic target for alopecia.

## Introduction

Hair loss is a widespread and progressive disorder that plagues a large number of people. The appearance of alopecia can be ameliorated by existing treatments, such as minoxidil and hair transplant surgery. However, the medications are only effective for mild-to-medium hair loss, and surgery is limited by the viability of hair follicles (HFs) in the donor scalp (Ho and Zito, 2019; Katzer et al., 2019).

Hair follicles are epidermal appendages that cycle between an active growth stage (anagen) and a quiescent stage (telogen), separated by an intermediate remodeling stage (catagen) (Plikus and Chuong, 2008). HF morphogenesis and cycling are regulated by complex and intricate interactions between the epithelial and mesenchymal cells, and require spatiotemporal integration of multiple stimulatory and inhibitory signals (Kishimoto et al., 2000; Baker and Murray, 2012; Veraitch et al., 2013; Kandyba and Kobielak, 2014). In this process, the dermal papilla (DP), a unique stem cell niche derived from the mesenchyme, serves as the signaling center that triggers hair cycling through a paracrine signaling mechanism (Inui and Itami, 2011; Leiros et al., 2012; Zhang et al., 2014). Extracellular vesicles (EVs) are thought to play a critical role in this process (Kwack et al., 2019; le Riche et al., 2019; Chen et al., 2020; Rajendran et al., 2020). These signal exchanges promote activation of cellular pathways that are essential for hair epithelial cell growth, differentiation, and function, such as inhibition and activation of the BMP/TGF-β and WNT signaling pathways (Jamora et al., 2003; Greco et al., 2009; Genander et al., 2014). However, cultured DPCs tend to lose their hair-inducing capacity during passage, and high-passage DPCs (≧P8) fail to trigger HFs when interacting with epidermal cells *in vivo* (Matsuzaki, 2008; Lin et al., 2016; Wang J. et al., 2018). Conditioned medium from low-passage DPCs, which contains DP-EVs, can stimulate DPC proliferation *in vitro* and mouse dorsal hair growth *in vivo* (Zhang et al., 2014, 2016). EVs secreted from DPCs can accelerate hair growth *in vivo* and *in vitro* (Zhou et al., 2018; Yan et al., 2019; Kwack et al., 2019; le Riche et al., 2019), but the mechanisms underlying this growth-promoting effect remain to be fully elucidated.

Extracellular vesicles are nano-vesicles secreted from various cell types that carry regulatory proteins, mRNAs, and microRNAs (miRNAs) (Mittelbrunn et al., 2011; Zhou et al., 2014). Several reports have documented the functional contributions of specific miRNAs to hair development, including miR-125b (Zhang et al., 2011), miR-24 (Amelio et al., 2013), miR-205 (Wang et al., 2013), and miR-31 (Mardaryev et al., 2010). Especially, a recent research has discovered that dermal exosomes containing miR-218-5p could promote hair regeneration by regulating β-catenin signaling in mice (Hu et al., 2020). These studies suggest that vesicular miRNAs might play an especially important role in governing cell-to-cell communication (Yu et al., 2016), silencing or promoting known genes and pathways related to HF regeneration, and could also serve as biomarkers (Carrasco et al., 2019; Yan et al., 2019). However, the specific miRNAs involved in this process are unclear, and very few studies have sought to identify the miRNAs in human DP-EVs by high-throughput RNA sequencing. In this study, we used a human scalp follicle organ culture model to explore the influence of low- and high-passage DP-EVs on hair growth and activation of lower outer root sheath cells (ORSCs) and hair matrix cells (MxCs). Using high-throughput miRNA sequencing, we selected the top 100 most differentially expressed miRNAs (DEMs) and analyzed the biological functions and networks of their predicted target genes. We identified miRNAs in DP-EVs that may be involved in the process of HF regeneration, providing insight into DP-EV–regulated HF regeneration at the molecular level. Quantitative real-time PCR (qRT-PCR), western blot, and immunofluorescence staining analysis of key target genes were also performed.

## Materials and Methods

### Isolation and Culture of Human HFs

Occipital scalp skin samples were obtained from discarded tissue from elective cosmetic operations performed on healthy adults (five women aged 35–45 years). Ethical approval and an institutional review board exemption were obtained from the Medical Ethical Committee of Southern Medical University.

Hair follicles in anagen stage were microdissected from each sample under an MZ8 dissecting microscope (Leica Microsystems, Wetzlar, Germany) (Langan et al., 2015). The basic culture medium (Corning Glassworks, Corning, NY, United States) containing 300 μl basic culture medium: William’s E medium supplemented with 10 ng/ml hydrocortisone, 10 μg/ml insulin, 10 U/ml penicillin, and 2 mM l-glutamine (Life Technologies, Paisley, United Kingdom) was supplemented with P3-DP-EVs (300 μg DP-EVs in 300 μl medium), P8-DP-EVs, or PBS (as a negative control). HFs were photographed using the Leitz Labovert inverted microscope (Leitz Labovert FS, Wetzlar, Germany), measured for length, and assessed for follicle bulb morphology every 24 h for 9 days. Unless specified, all materials were supplied by Sigma-Aldrich (Dorset, United Kingdom).

### Preparation of Cells and Isolation of DP-Derived EVs

Human scalp follicles were separated, and hair bulbs were cut and collected using microsurgical scissors and microscope forceps under the microscope. Hair bulbs were incubated with 0.2% collagenase I (Sigma-Aldrich) in PBS for 1 h at 37°C. The pellet was cultured in DMEM containing 10% FBS in 5% CO_2_ at 37°C. Media were collected from low (P3) and high (P8) passage hDPC cultures and stored at −20°C. EVs were isolated by ultracentrifugation (SW 32 Ti swinging-bucket rotor in a Beckman Coulter L8-80M Ultracentrifuge) as described previously (Rajendran et al., 2020). In brief, 500 ml hDPC medium was centrifuged at 300 × *g* for 10 min and 2000 × *g* for 10 min to remove live cells and dead cells, respectively. The supernatant was then filtered with pressure through 0.22 μm membrane filters to remove cell debris and vesicles > 220 nm in diameter. Next, the supernatant was ultracentrifuged at 100,000 × *g* for 70 min. Finally, the pellet was washed and resuspended in PBS and stored at −80°C. The negative control was an equal volume of 1 × PBS, which was prepared in the same way as the actual EV samples.

Outer root sheath cells from the lower outer root sheath region were isolated and prepared as described previously (Lü et al., 2006; Zhou et al., 2018), the single-cell suspension was collected and cultured in Defined Keratinocyte serum-free medium (DK-SFM) (Gibco) at 37°C. Human MxCs were purchased from ScienCell Research Laboratories (ScienCell, San Diego, CA, United States; Cat. #2410) as previously described (Chen et al., 2020). As recommended by the provider, MxCs were cultured in MSC medium (ScienCell, Cat. #7501) with 5% exosome-free FBS, 1% stem cell growth supplement (ScienCell, Cat. #7562), and 1% penicillin–streptomycin. Cells were incubated in an atmosphere containing 5% CO_2_ at 37°C.

### Verification of DP-EVs

The particle size of DP-EVs was investigated by nanoparticle tracking analysis (NTA) on a NanoSight NS300 (Malvern Panalytical, Malvern, United Kingdom). Before acquisition, the highly concentrated samples were diluted in Milli-Q water (1:1000). Three individual measurements were obtained and analyzed using the NTA software version 2.3 (Malvern Panalytical).

Transmission electron microscopy (TEM) was used to characterize the morphology of DP-EVs. Briefly, DP-EVs were fixed in 3% glutaraldehyde for 2 h and washed twice with PBS. Ten micrograms of EV suspension was loaded onto formvar carbon-coated 200 mesh copper grids for 20 min at room temperature. Adsorbed DP-EVs were negatively stained with 1% phosphotungstic acid for 5 min. The grids were then washed three times with distilled water and examined on an H-600 transmission electron microscope (Hitachi, Tokyo, Japan) at 100 kV.

For DP-EV labeling and uptake assay, a 100 μl suspension of EVs was labeled using the PKH26 Red Fluorescent Cell Linker Kit for General Cell Membrane Labeling (DiI; Sigma-Aldrich, St. Louis, MO, United States) with a 20 min incubation at room temperature. To wash out the dye, the sample was ultracentrifuged twice at 100,000 × *g* for 1 h. As a negative control, an equal volume of PBS supernatant was mixed with DiI and then prepared in the same way as the actual exosome samples. The precipitate was resuspended in 100 μl PBS. For the DPC uptake assay, an appropriate amount of DP-EVs (as determined by BCA protein assay) was added to the cell culture. P3 lower ORSCs and MxCs were transfected with GFP and cultured with DiI-labeled DP-EVs for 24 h. Cells were fixed with 4% paraformaldehyde for 15 min and washed three times with PBST, and then the coverslips were mounted on glass slides using ProLong Diamond Antifade Mountant with DAPI (Life Technologies/Thermo Fisher Scientific, Waltham, MA, United States) prior to imaging on a confocal laser scanning microscope (LSM510; Carl Zeiss, Jena, Germany).

### Western Blot Analysis

For identification of vesicular markers, protein extracts were isolated from EVs using RIPA protein lysis buffer containing 1 mM PMSF. Equal amounts of protein (10 μg/lane) were subjected to SDS-PAGE under non-reducing conditions, and the gels were blotted onto nitrocellulose membranes (Pierce, Waltham, MA, United States) in an electrophoretic transfer cell (Bio-Rad). The membrane was blocked with 5% non-fat milk at 4°C overnight and then probed with anti-CD63 (1:800 dilution; Abcam), anti-CD9 (1:800 dilution; Abcam), and anti-ALIX (1:800 dilution; Abcam) antibody for 1 h. The membrane was incubated with sheep anti-mouse IgG conjugated to HRP (NeoBioscience, Shenzhen, China) for 1 h. Band density was determined using the Bio-Rad Quality One Software. To detect the expression of key proteins in ORSCs and MxCs, total protein was extracted from human HF using RIPA lysis buffer and subjected to western blot analysis as described above. Antibodies against the following proteins were obtained from Abcam (Cambridge, United Kingdom): β-catenin (1:100 dilution), Lef-1 (1:100 dilution), Shh (1:100 dilution), Gli1 (1:100 dilution), Cyclin D1 (1:400 dilution), Cyclin E (1:400 dilution), BMP2 (1:800 dilution), and SMAD5 (1:400 dilution). p-SMAD5 was obtained from Millipore (1:800 dilution).

### Histological Examination and Immunofluorescence Staining

Grafted follicles were taken on days 1, 10, 15, 20, and 30 after transplantation and embedded in paraffin blocks. Five micron-thick longitudinal sections were prepared, followed by hematoxylin and eosin (HE) staining. Images were taken by optical microscope. Follicle specimens were fixed in 4% paraformaldehyde at 4°C for 12 h. Following antigen retrieval using citrate buffer, the sample was blocked with 5% serum. Images were obtained on an IX61 FL fluorescence microscope (Olympus) and analyzed using Image-Pro Plus. Images were also obtained using an Olympus FLUOVIEW FV10i confocal laser scanning microscope. Eight images were taken of each sample, and a normalized average-intensity picture of images was used for analysis. Images were analyzed using the Image-Pro Plus software.

### Processing of RNA-Seq Data, Target Gene Prediction and Functional Enrichment

Total miRNAs from DP-EVs (P3 and P8) were extracted and subjected to miRNA sequencing. RNA quality was verified on an Agilent 2100 BioAnalyzer (Agilent Technologies, Santa Clara, CA, United States). Quality control of raw sequence data was performed using the FastQC tool. RNA expression levels were expressed as reads per kilobase per million (RPKM) mapped reads. The DESeq package was used to detect DEMs between the DP-EVs (P3) and DP-EVs (P8). Genes with RPKM > 1 were included in the subsequent analyses. The threshold was set to a twofold change and an FDR < 0.05. The top 100 differentially expressed DEMs were visualized on a heatmap. The heatmap and unsupervised cluster analysis of the top 100 DEMs were plotted using *z*-score normalization on OmicShare tools, an online platform for data analysis^1^. Target genes of DEMs were predicted using TargetScan^2^ and miRDB^3^ (Agarwal et al., 2015). Using OmicShare tools, GO analysis was performed to elucidate the concrete biological functions of specific genes (Ashburner et al., 2000), and KEGG pathway enrichment was used to identify the critical signal pathways (Kanehisa et al., 2017). To further elucidate the molecular mechanisms related to the predicted genes, TargetScan, miRanda, PicTar, PITA and DIANA-microT databases (Krek et al., 2005; Paraskevopoulou et al., 2013; Vejnar et al., 2013; Agarwal et al., 2015; Wong and Wang, 2015) were used in combination to predict the miRNAs responsible for regulating the predicted hub genes.

To screen PPIs (protein–protein interaction), the target genes of DEMs were mapped to the STRING database^4^ (Franceschini et al., 2013), and networks were visualized using Cytoscape (Smoot et al., 2011). The most significant modules were identified with the plug-in MCODE of Cytoscape with a cutoff MCODE score of >5 (Bandettini et al., 2012). Hub genes were identified using the CytoHubba plug-in of Cytoscape, ranked by Maximal Clique Centrality (Chin et al., 2014). HF-related miR-140-5p targets were identified using TargetScan and based on their co-occurrence with the search term “hair follicle signaling” in the PubMed database (Liu et al., 2008; Rishikaysh et al., 2014; Akilli et al., 2015; Yi, 2017). Interactions between network genes were annotated using Cytoscape.

### Dual-Luciferase Reporter Assay

For the purpose of verifying the binding relationship between miR-140-5p and BMP2, and to assess whether BMP2 is a direct target gene of miR-140-5p, ORSCs were seeded in 6-well plates (Guangzhou Jet Bio-Filtration Co., Ltd, TCP-010-006) at a density of 2.5 × 10^5^ cells/well with the complete media. The artificially synthesized 3′-UTR of BMP2 was inserted into the pMIR-reporter vector (Yue Yang Biotechnology Co., Ltd., Beijing, China). A site-specific mutation was then introduced into the 3′-UTR fragment. One microgram of BMP2-wild type (WT) or BMP2-mutant type (MUT) was cotransfected into ORSCs with 50 nM miR-140-5p mimic or miR-NC. Transfections were performed using Lipofectamine 2000 (Invitrogen) with Opti-MEM (Thermo Fisher Scientific). After 48 h, the cells were lysed and subjected to luciferase assay using the dual-luciferase reporter assay reagent (GeneCopoeia, Rockville, MD, United States). Luciferase activity was detected using the Luciferase Assay kit (K801-200; Biovision, San Francisco, CA, United States) on a Glomax20/20 luminometer (Promega Corporation, Madison, WI, United States). The 3′-UTR of BMP2 was obtained from GeneCopoeia.

### RNA Interference

The concentration of transfection units per milliliter (TU/mL) was calculated using the following formula: (% infected cells × cells used in the titration/100 × 1000 μl/μl virus added to the well) = TU/mL. The stable overexpression and inhibition of miR-140-5p in P3-DPCs were established by stable transduction with lentivirus (1E + 8 TU/mL) (Sino Biological) at a multiplicity of infection (MOI) of 10. In brief, DPCs (P3) were transfected with miRNA oligonucleotides containing an 50 nM miR-140-5p overexpression sequence and the corresponding negative control vector (miRNA-NC), or an 50 nM miR-140-5p inhibiting segment (si-miR-140-5p) and the corresponding negative control vector (si-miRNA-NC), all purchased from Sino Biological. In both the SiHa and C33a cell lines, the transfected cells were designated as miRNA-NC, miR-140-5p, si-miRNA-NC, and si-miR-140-5p, and DPCs treated with an equal volume of PBS were used as a blank control. After 12 h of culture, EVs (miRNA-NC-EV, miR-140-5p-EV, si-miRNA-NC-EV, and si-miR-140-5p-EV) were isolated from DPCs and used for subsequent experiments. An equal amount of PBS was used as a blank control.

### Animal Model Experiments

All animal experiments were performed using 7-week-old female C57BL/6 mice. Animals were purchased from the Experimental Animal Centre at Southern Medical University (Guangzhou, China). Depilated mice were randomly injected with miRNA-NC-EVs, miR-140-5p-EVs, si-miRNA-NC-EVs, or si-miR-140-5p-EV (4 mg/ml dissolved in PBS, 1 ml per mouse) every 2 days, or PBS without EVs (as a negative control). Ethical approval for all experimental procedures was obtained from the Experimental Animal Centre at Southern Medical University.

### Quantitative (q)RT-PCR

Total RNA from regenerative HFs was isolated using the Trizol reagent (Life Technologies), and cDNA was generated using the PrimeScript RT-PCR Kit (Takara, Dalian, China). qRT-PCR was performed using the SYBR PrimeScript RT-PCR Kit (Takara), Power SYBR Green PCR Master Mix (Life Technologies), and ABI Prism 7900HT Sequence Detection System (Life Technologies). PCR cycling conditions were as follows: denaturation for 10 min at 95°C, followed by 40 cycles of denaturation (95°C for 15 s), annealing (60°C for 20 s), and extension (72°C for 10 s). Fold-changes in relative gene expression were calculated using the 2^–Δ^ ^Δ^ ^*Ct*^ method. For miRNA quantification, total RNA was reverse-transcribed using the TaqMan advanced miRNA cDNA synthesis kit (Applied Biosystems). All specific primers for gene and miRNA expression were purchased from Applied Biosystems. Primer sequences used in this study are provided in Supplementary Table S1.

### Statistical Analysis

Statistical analysis was performed using SPSS 16.0 software (SPSS, Inc, Chicago, IL, United States). All values are shown as means ± SD ANOVA was used for multiple comparisons. Comparisons between groups were performed using the Wilcoxon test and *t*-test. *p* < 0.05 was considered statistically significant. Each experiment was repeated at least three times. For statistical assessment of cultured HFs, the mean value per donor under each treatment was determined before calculation of the sample mean, data from each experimental condition were analyzed for normal distribution using the Kolmogorov–Smirnoff test.

## Results

### Difference Between Low- and High-Passage DPCs

To determine the biological difference between low- and high-passage DPCs, we isolated DPCs from human follicle dermal fibroblasts for culturing, and collected cells in P3 and P8 for observation. P3-DPCs exhibited the tendency to aggregate, whereas P8-DPCs lost this property (Supplementary Figure S1A). To characterize intrinsic properties relevant to HF inductivity in DPCs, distinct DP markers including alkaline phosphatase (ALP), neural cell adhesion molecule (NCAM), and α-smooth muscle actin (α-SMA) were monitored by IF, western blot, and RT-PCR analysis (Lin et al., 2016; Wang J. et al., 2018) (Supplementary Figures S1B–E). As illustrated in Supplementary Figure S1, expression of ALP and NCAM was higher in P3-DPCs than in P8-DPCs (^∗∗^*p* < 0.05), but expression of α-SMA increased with increasing passage number. The results indicated that the molecular characteristics of human DPCs differed between high and low passage.

### Preparation and Characterization of DP-EVs

**FIGURE 1:**
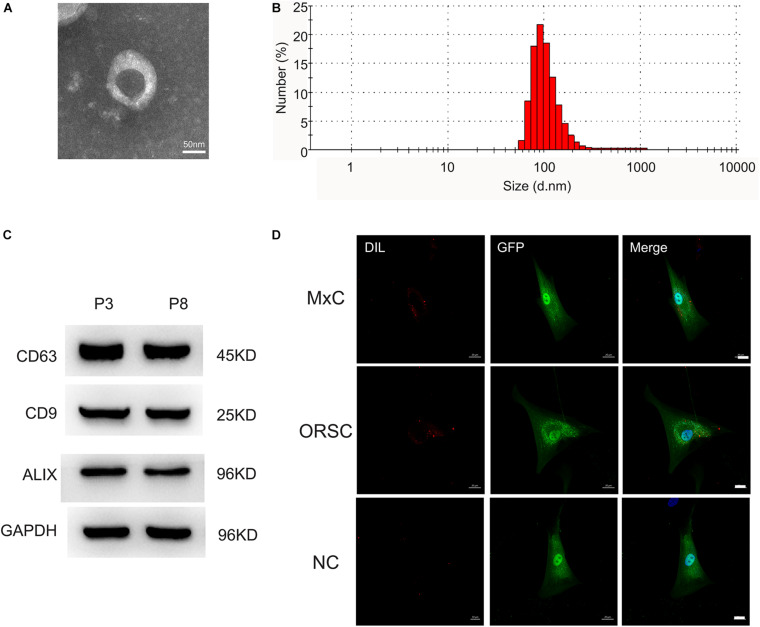
Characterization of DP-EVs from P3 and P8 DPCs. **(A)** Transmission electron microscopy morphology of DP-EVs. **(B)** Vesicular size distribution, measured by NTA. Scale bar: 50 nm. **(C)** Western blots of DP-EVs to detect CD63, CD9, and ALIX; *n* = 8 for each group. **(D)** DP-EVs were labeled with DiI and taken up by GFP-transfected MxCs and ORSCs. As a negative control, an equal volume of PBS mixed with DIL was cultured with ORSCs. *n* = 8 for each group. Scale bar: 50 μm.

Extracellular vesicles were isolated from low-passage (P3) and high-passage (P8) human DPCs by ultracentrifugation. The morphology of DP-EVs was analyzed by TEM, which revealed that they had a cup- or round-shaped form (Figure 1A). NTA revealed that the diameter of DP-EVs ranged from 58.8 to 255.0 nm, with an average diameter of 91.3 ± 18 nm (Figure 1B). Western blots showed that DP-EVs expressed EV-specific surface markers such as CD63, CD9, and ALIX (Figure 1C). To monitor the uptake of DP-EVs, we applied P3 human MxCs and ORSCs transfected with GFP, and labeled DP-EVs with DiI (red). Cells were cultured with DP-EVs for 24 h. Under the fluorescence microscope, DiI-labeled EVs were observed in the cytoplasm of MxCs and ORSCs (Figure 1D), whereas ORSCs in the negative control group had no DiI signaling in the cytoplasm (Figure 1D). These data suggested that we successfully isolated DP-EVs, which could be absorbed by MxCs and ORSCs, respectively.

### The Therapeutic Effect of DP-EVs on Human HF Growth in Organ Culture

**FIGURE 2:**
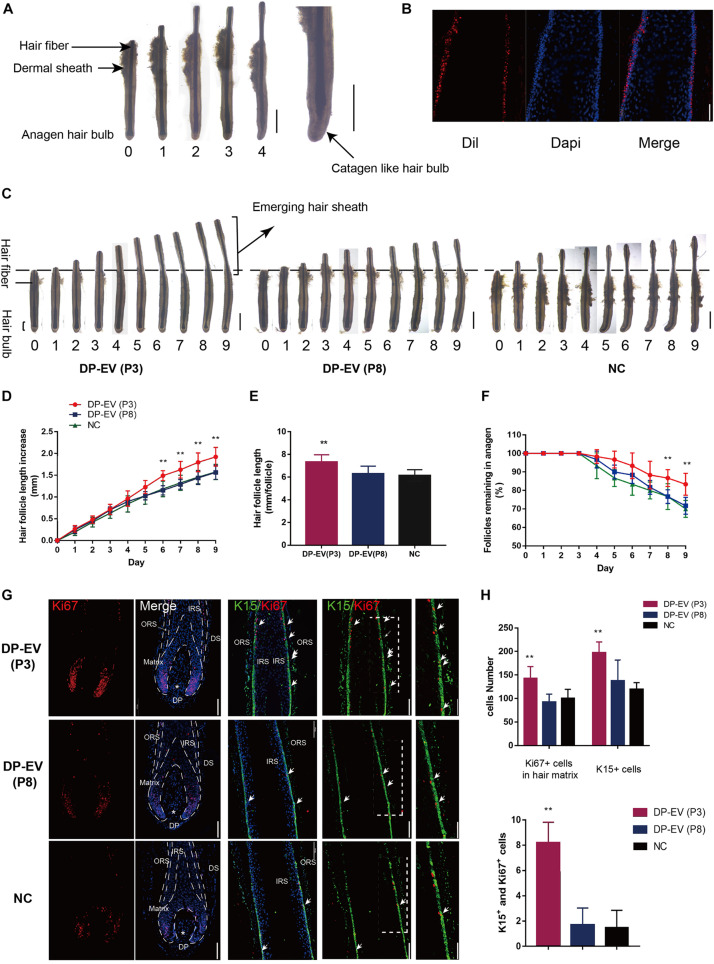
The therapeutic effect of DP-EVs on human scalp HF growth in organ culture. **(A)** Sequential photomicrographs, taken every 24 h, of individual scalp follicles in organ culture under the indicated conditions. Scale bars: 1 mm. Some hair follicles exhibited a catagen-like morphology by day 4. Scale bars: 0.5 mm. **(B)** DiI-labeled DP-EVs were absorbed by HF lower ORS. Scale bar: 20 μm. **(C)** Hair follicles were cultured with P3- or P8-DP-EVs (containing 300 μg DP-EVs in 300 μl culture medium) or negative control medium. Scale bars: 1 mm. **(D–F)** Hair follicles in each group were assessed and measured daily for increase in hair length **(D)**, accumulated hair length **(E)**, and the percentage of hair follicles remaining in anagen **(F)**. **(G)** Immunofluorescence staining of Ki67 and K15 in the hair matrix and lower ORS region. Scale bar: 100 μm. **(H)** Quantitative analysis of the number of Ki67+, K15+, and Ki67+/K15+ double-staining cells in hair follicle. Data are expressed as means ± SD. *n* = 60 from give healthy female individuals (12 samples from each individual). **p < 0.01 vs. PBS-treated group (NC). Statistical significance determined by one-way ANOVA with Bonferroni comparisons; *n* = 5 for each group.

To determine whether DP-EVs could affect scalp HF growth, we grew isolated HFs in basic culture medium supplemented with various concentrations of DP-EVs and collected daily images to assess their morphology. HFs in basic culture medium produced hair fiber, inner root sheath (IRS), and ORS, but not the dermal sheath (Figure 2A). By day 4, some HFs exhibited a catagen-like morphology: pigmentation had ceased, and the DP was detached from the hair fiber and hair matrix (Figure 2A). Treatment with 0.01–1 μg/μl P3-DP-EV enhanced HF growth in a dose-dependent manner (Supplementary Figure S2). Treatment with 1 μg/μl DP-EVs had a significantly stronger anagen-prolonging effect than lower concentrations (0.1 and 0.01 μg/μl). Hence, we chose 1 μg/μl DP-EVs for subsequent experiments. We previously reported that DiI-labeled DP-EVs were absorbed by hair matrix (Chen et al., 2020). However, we found that DiI-labeled DP-EVs were also absorbed and integrated into the lower ORS region (Figure 2B).

To assess the therapeutic effect of DP-EVs in organ culture, we added DP-EVs from P3 and P8 DPCs to cultured human HFs; HFs treated with PBS were used as a negative control (Figure 2C). EVs from P3 DPCs accelerated human hair growth and prolonged hair anagen (Figures 2C–F). By day 9, the length of HFs cultured with DP-EVs (P3) increased by 1.93 ± 0.12 mm, whereas HFs cultured with DP-EVs (P8) and PBS increased by only 1.57 ± 016 mm and 1.58 ± 0.18 mm, respectively (Figure 2D). Following treatment with P3-DP-EVs, about 83.4% of HFs were in anagen on day 9, about 13% higher than in samples treated with DP-EVs (P8) or PBS (Figure 2F). We also assessed cell viability of human HFs on day 5 based on Ki67 immunofluorescence. The results revealed that HFs treated with DP-EVs (P3) had the most Ki67-positive cells in lower ORS and hair matrix (*p* < 0.01) (Figures 2G,H), indicating that HFs treated with low-passage DP-EVs significantly activated hair growth. Combined with the fluorescence staining results (Figure 2B), these observations indicate that EVs can be absorbed by HFs in the lower ORS and hair matrix region, and can activate proliferation of lower ORSCs and MxCs. We then performed double staining for K15 and Ki67. In the lower ORS region, K15 is highly expressed (Moll et al., 2008). Immunofluorescence staining revealed significant widening of the K15+-stained lower ORS region at day 5 in HFs treated with DP-EVs (P3) (Figures 2G,H), indicating that low-passage DP-EVs stimulated ORS cell proliferation in cultured human HFs.

### Effect of DP-EVs on Human ORSCs

**FIGURE 3:**
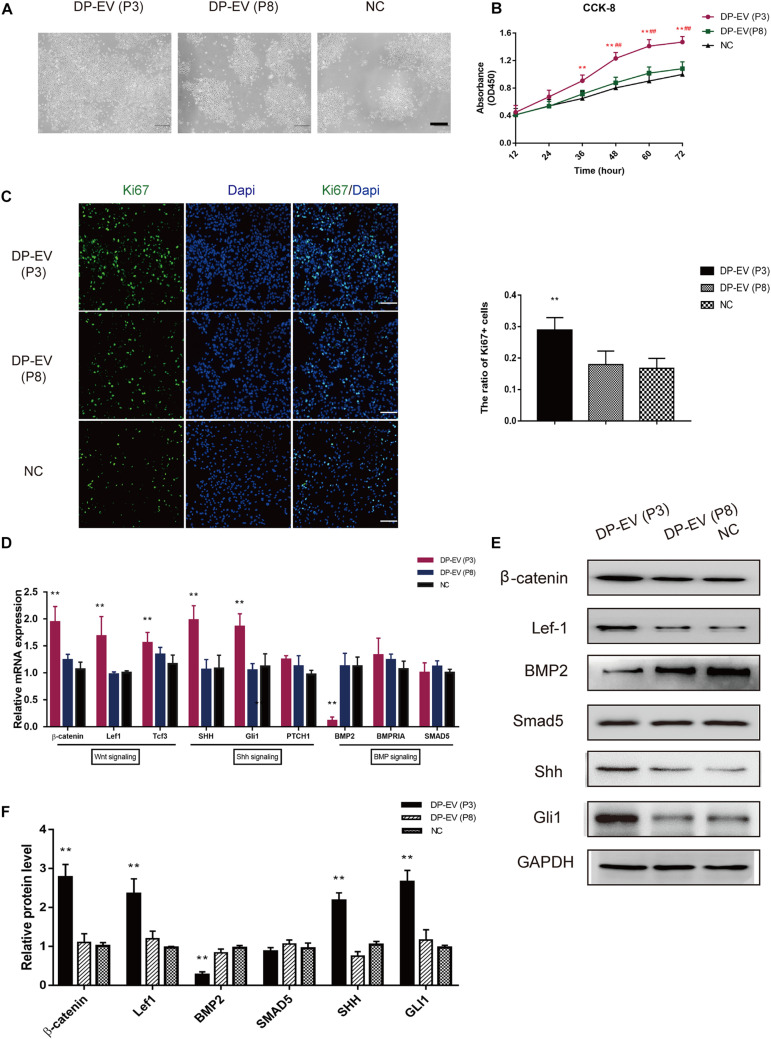
Effect of DP-EVs on human hair follicle lower outer root sheath cells (ORSCs). **(A,B)** ORSCs were treated with DP-EVs from P3 and P8 human DPCs, and cell viability was examined by CCK-8 assay. **(C)** Immunofluorescence microscopy and quantitative analysis of the percentage of Ki67 + ORSCs treated with DP-EVs (P3), DP-EVs (P8), or PBS for 48 h. Scale bar: 100 μm. **(D)** RT-PCR analysis of known follicle-regulatory genes in ORSCs after DP-EV treatment for 5 days. **(E,F)** Western blot analysis of known follicle-regulatory proteins in ORSCs after DP-EV treatment for 5 days. Data are expressed as means ± SD. **p < 0.01 vs. negative control (NC); ^##^*p* < 0.01 vs. P8-DP-EV–treated group. Statistical significance was evaluated by one-way ANOVA with Bonferroni comparison; *n* = 5 for each group.

In a previous study, we showed that low-passage DP-EVs significantly promoted MxC proliferation (Chen et al., 2020). After treating lower ORSCs with low- or high-passage DP-EVs, we assessed their proliferation (Figure 3A). The results of CCK-8 and Ki67 immunofluorescence staining revealed that low-passage DP-EVs promoted cell proliferation, with a higher Ki67^+^ cell ratio than P8-DP-EV treatment (Figures 3B,C).

We previously reported the stimulatory effect of MxCs, as well as inhibition of BMP signaling and promotion of Wnt signaling by DP-EVs (Chen et al., 2020). To investigate changes in hair growth regulators of ORSCs, we evaluated the expression levels of known follicle-regulatory genes in the Wnt, BMP, and Shh signaling pathways in ORSCs (Watt et al., 2008; Choi, 2018) by RT-PCR and western blot (Figures 3D–F). As shown in Figure 3D, at day 5 in cells treated with DP-EVs (P3), hub genes in the Wnt signaling pathway, including β-catenin, Lef-1, and Tcf3, were upregulated, whereas BMP2 was downregulated. Shh and Gli1 in the Shh signaling pathway were also upregulated (Figure 3D). In accordance with the results of RT-PCR, levels of components of the Wnt (β-catenin and Lef-1) and Shh signaling pathways (Shh and Gli1) increased after 5 days of low-passage DP-EV treatment, whereas the BMP2 level significantly decreased. These data indicated that low-passage DP-EVs may promote ORSC proliferation by up-regulating the Wnt and Shh pathways and down-regulating the BMP2 pathway.

### Identification and Validation of Differentially Expressed miRNAs in Low- and High-Passage DP-EVs

**FIGURE 4:**
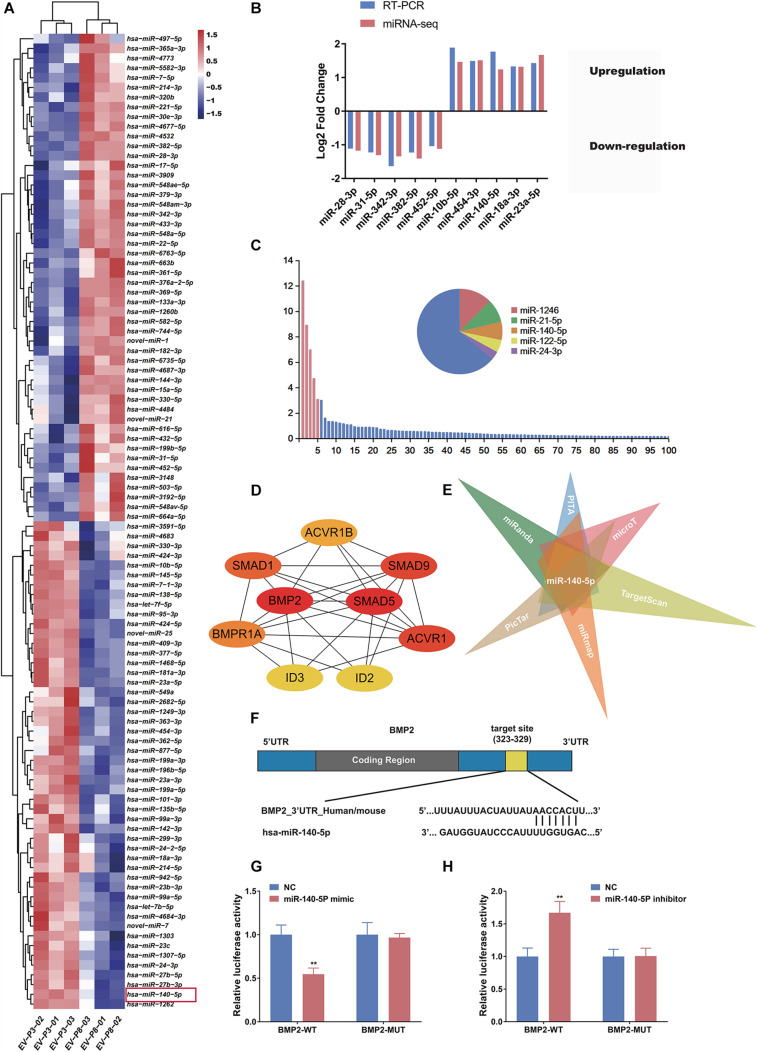
miR-140-5p–enriched DP-EVs decrease the expression of BMP2. **(A)** Heatmap and unsupervised cluster analysis of DEMs between DP-EVs (P3) and DP-EVs (P8). RNA expression level is represented by colors: bright red indicates high values, and blue indicates low values. **(B)** Validation of RNA-Seq data. **(C)** Relative proportion of miRNAs in total miRNA reads of DP-EVs (P3). U6 was used as an endogenous control; *n* = 8 for each group. **(D)** The most significant module obtained from the PPI network had 9 nodes and 28 edges. Hub genes (*BMP2* and *SMAD5*) were identified by Cytohubba. **(E)** Potential miRNAs involved in regulation of BMP2, predicted using the TargetScan, miRanda, miRmap, PicTar, PITA, and DIANA-microT databases. **(F)** Schematic diagram of miR-145 binding site on the 3′UTR of *SMAD5* mRNA, according to the TargetScan website. **(G,H)** Transfection of ORSCs with 50 nM miR-140-5p mimic **(G)** and inhibitor **(H)** for 48 h altered luciferase activity of wild-type and mutant *BMP2* 3′ UTR construct. Error bars denote the standard error of the means of three experiments. **p < 0.01 (two-tailed Student’s *t*-test).

Extracellular vesicles deliver various miRNAs to other cells and affect cellular functions (Witwer et al., 2013; Cobelli et al., 2017). Several lines of evidence suggest that miRNA might play a more important role in cell-to-cell communication (Carrasco et al., 2019; Hu et al., 2020), acting as promoters or suppressors by silencing or promoting known genes and pathways related to HF growth. To determine how low-passage DP-EVs augment hair growth, we deduced that the miRNAs in DP-EVs may be delivered to hair matrix and lower ORS to activate them and promote their proliferation. Therefore, we investigated the miRNA expression profiles of EVs derived from human DPCs at P3 and P8 by high-throughput sequencing; the datasets have been deposited in the Gene Expression Omnibus (GEO) database^5^. Of the 563 hsa-miRNAs screened (Supplementary Table S2), we identified the top 100 most DEMs (i.e., 50 upregulated and 50 downregulated) in P3 vs. P8 DP-EVs (cutoff: *p* < 0.05; fold change > 2.0) (Supplementary Table S3). To highlight differences between the two groups, we then performed an unsupervised clustering analysis of the 100 DEMs (Figure 4A). In this analysis, relationships among genes and samples are represented by trees whose branch lengths reflect the degree of similarity between the variables. To validate the profiling data, we performed RT-PCR on the same RNA samples used for the miRNA-Seq and confirmed the altered expression of 10 randomly selected miRNAs that were strongly up- or downregulated. Consistent with the miRNA-Seq data, the results showed that miR-10b-5p, miR-454-3p, miR-140-5p, miR-18a-3p, and miR-23a-5p were upregulated and miR-28-3p, miR-31-5p, miR-342-3p, miR-382-5p, and miR-452-5p were downregulated in low-passage vs. high-passage DP-EVs (*p* < 0.05) (Figure 4B). In P3 DP-EVs, the five most abundant miRNAs (miR-1246, miR-21-5p, miR-140-5p, miR-122-5p, and miR-24-3p) accounted for 36.0% of the total miRNA reads (Figure 4C), and miR-140-5p and miR-24-3p were upregulated.

### Identification of Target Genes and Functional Analysis

**FIGURE 5:**
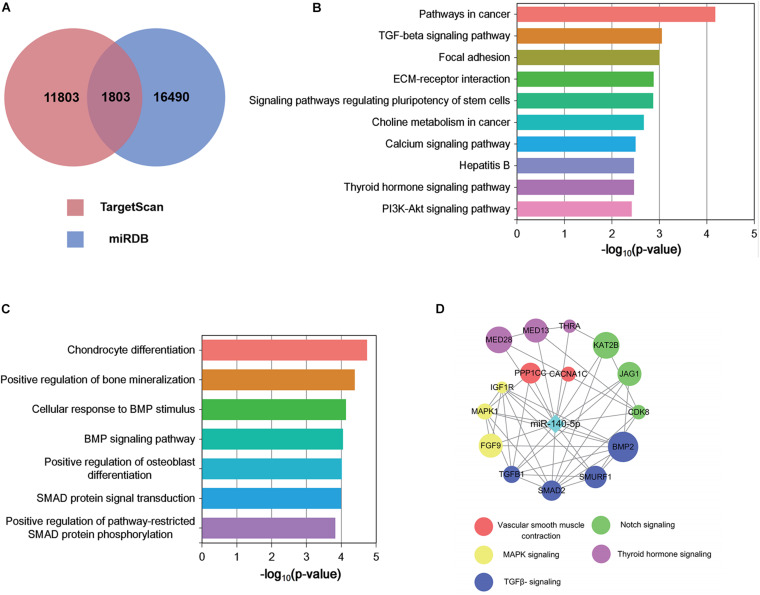
Functional analysis of predicted miRNA target genes. **(A)** Target genes of miRNAs predicted by TargetScan and miRDB. **(B,C)** KEGG **(B)** biological process and GO pathway enrichment **(C)** analysis of the predicted target genes of differentially expressed miRNAs. **(D)** Predicted direct targets of miR-140-5p that are related to hair follicles.

Each microRNA can regulate a large number of target genes, and several databases based on various algorithms are available for prediction of miRNA gene targets. We chose TargetScan and miRDB to predict the targets of the 100 DEMs, resulting in the identification of 1803 genes (Figure 5A and Supplementary Table S4). To analyze the biological classifications of the predicted target genes, we performed functional and pathway enrichment analyses. Using KEGG pathway analysis to mine canonical signaling pathways, we found that the predicted target genes were strongly associated with the TGF-β Signaling Pathway (Figure 5B and Supplementary Table S5) (BMP belongs to the TGF-β family). GO analysis revealed that changes in the biological processes (BP) of the predicted target genes were significantly associated with GO terms such as “chondrocyte differentiation,” “BMP signaling pathway,” and “positive regulation of bone mineralization” (Figure 5C and Supplementary Table S6). To explore interactions among the predicted target genes, we constructed a PPI network of the target genes and screened the most important modules using MCODE. The significant module with the highest score (7.75) included 9 nodes and 28 edges (Figure 4D). Genes often interact with each other through hub genes; indeed, CytoHubba identified BMP2 and SMAD5 as the most significant hub genes involved in this module (Figure 4D). As above, we focused subsequent studies on the BMP signaling pathway.

### miR-140-5p Targets BMP2 and Negatively Correlates With BMP2

The screen identified BMP2 and its downstream target SMADs as the hubs of the regulated gene module (Figure 4D), and BMP2 was downregulated in ORSCs treated with low-passage DP-EVs, whereas expression of SMADS remained unchanged (Figures 3D–F). Hence, to explore the molecular mechanisms accounting for the BMP2-suppressive role of miRNAs in DP-EVs, we screened for miRNAs that regulate BMP2 using the PITA, miRmap, microT4, miRanda, PicTar, and TargetScan databases (Supplementary Table S7). The results (Figure 4E) revealed that among the 100 DEMs, only miR-140-5p (accounting for 6.96% of the total miRNA reads in passage 3 DP-EVs in Figure 4C) was scored as a potential regulator of BMP2 in all databases. The TargetScan website revealed the target binding sequences of miR-140-5p in the BMP2 mRNA (Figure 4F). In addition, we constructed an interaction network of miR-140-5p and its target genes (Supplementary Table S8). To refine the list of potential target genes downstream of miR-140-5p that might be specifically involved in HF growth, we identified targets of miR-140-5p within a HF-related gene network (Figure 5D). The predicted miR-140-5p network includes key genes that regulate multiple points in the TGF-β superfamily (to which BMP belongs), and the sizes of the nodes represent the binding score of miR-140-5p to target genes. Regulation of a target mRNA by an miRNA requires temporal and spatial coexpression. Therefore, we transfected ORSCs with a plasmid containing a luciferase-BMP2 3′ UTR construct to test the predicted miR-140-5p-target interactions. The luciferase reporter assay showed that co-transfection of miR-140-5p mimic or inhibitor along with the wild-type BMP2 reporter caused significant down-regulation or up-regulation, respectively, of luciferase reporter activity (*p* < 0.01) (Figures 4G,H). However, co-transfection of miR-140-5p mimic or inhibitor along with the mutant BMP2 reporter had no effect on reporter activity (Figures 4G,H). These results indicated that miR-140-5p in low-passage DP-EVs inhibits the expression of BMP2 in ORSCs.

### DPC-Derived Vesicular miR-140-5p Promotes ORSC and MxCs Proliferation *in vitro*

**FIGURE 6:**
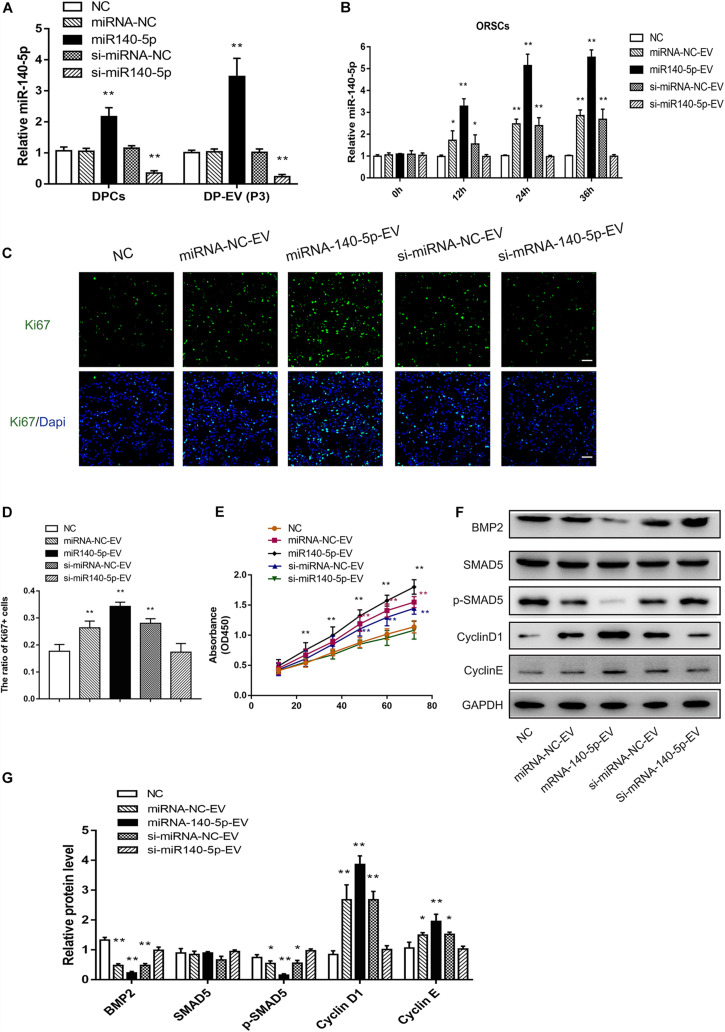
Effect of DPC vesicular miR-140-5p on ORSCs *in vitro*. DP-EVs were isolated from DPCs overexpressing (miR-140-5p) or inhibiting miR-140-5p (si-miR-140-5p) for 48 h (concentration for miRNA mimics or inhibitor: 50 nM). The control group was transfected with an equal volume of control lentiviral vector (miR-NC, si-miR-NC) or PBS. **(A)** RT-PCR was performed to analyze the relative expression of miR-140-5p in DPCs and their EVs. **(B)** ORSCs were treated with DP-EVs for the indicated lengths of time (0, 12, 24, and 36 h), and then miR-140-5p was detected by RT-PCR. **(C,D)** Immunofluorescence microscopy and quantitative analysis of the percentage of Ki67 + ORSCs treated with DP-EVs or PBS for 48 h. Scale bar: 50 μm. **(E)** CCK-8 assay for cell viability of ORSCs treated with DP-EVs at the indicated time points. **(F,G)** Western blot and quantification of BMP regulatory proteins and cyclins in ORSCs after DP-EV treatment for 5 days. Data represent means ± SD of triplicates (*p < 0.05; **p < 0.01).

Based on the bioinformatics analysis findings, we examined the pro-proliferation effect of DPC-derived vesicular miR-140-5p in ORSCs. First, we investigated whether EVs transferred miR-140-5p into ORSCs. To this end, we isolated overexpressing or inhibiting miR-140-5p DPCs established by lentivirus transfection. Real-time PCR analysis revealed an elevated miR-140-5p expression level in miR-140-5p–overexpressing DPCs, and a reduced miR-140-5p level in miR-140-5p–silenced (si-miR-140-5p) DPCs, relative to control cells (DPCs transfected with miR-NC or si-miR-Nc) and parental cells (Figure 6A; ^∗∗^*p* < 0.01). A similar tendency was observed in EVs derived from DPCs (Figure 6A; ^∗∗^*p* < 0.01). To confirm the role of EVs in transporting miR-140-5p to ORSCs, we incubated ORSCs miR-140-5p–overexpressing or –inhibited DP-EVs, and harvested the cells for qRT-PCR analysis at various times (0, 12, 24, and 36 h). As shown in Figure 6B, miR-140-5p levels in ORSCs were markedly upregulated after the cells were treated with miRNA-NC, miR-140-5p–overexpressing, or si-miRNA-NC DP-EVs for 12 h, but were most upregulated in miR-140-5p–overexpressing DP-EVs, indicating that DP-EVs transferred miR-140-5p into ORSCs. However, this effect was abolished by miR-140-5p silencing. ORSCs treated with an equal amount of PBS was used as a negative control. After 12 h of culture, EVs derived from the transfected DPCs were designated as miRNA-NC-EVs, miR-140-5p-EVs, si-miRNA-NC-EVs, or si-miR-140-5p-EVs and used for subsequent experiments.

To investigate the function of DPC-derived vesicular miR-140-5p in ORSCs, we examined the pro-proliferation activity of DP-EVs by Ki67 and CCK-8 assay in ORSCs (Figures 6C–E). As illustrated in Figure 6C–E, ORSCs treated with miR-140-5p–overexpressing EVs had the highest Ki67^+^ cell ratio and maximum absorbance at 450 nm at 48 h (Figures 6C–E; ^∗∗^*p* < 0.001) (40 μg EVs were added to the medium on day 0 and day 3). The pro-proliferation effect of DP-EVs was eliminated in the miR-140-5p–silence group. These results indicated that vesicular miR-140-5p stimulates ORSC proliferation.

By bioinformatics analysis and luciferase activity assay (Figures 4G,H), we identified BMP2 as the most probable downstream target gene of miR-140-5p. To further confirm the regulatory effect of vesicular miR-140-5p on BMP2, we evaluated the expression of BMP2 and its downstream targets SMAD5 and p-SMAD5 in ORSCs treated with various DP-EVs. Western blot revealed that overexpression of miR-140-5p led to a reduction in the levels of BMP2 and p-SMAD5, but not SMAD5. By contrast, inhibition of miR-140-5p markedly reversed the DP-EV–induced reduction of BMP2 and p-SMAD5 in ORSCs (Figures 6F,G). The result also showed that ORSCs treated with miR-140-5p–overexpressing EVs on day 5 expressed the highest levels of Cyclin D1 and Cyclin E, which are key cell cycle-related proteins, suggesting that miR-140-5p–overexpressing EVs affected ORSC cell proliferation. By contrast, cells treated with miR-140-5p–silenced EVs expressed the lowest levels of Cyclin D1 and Cyclin E (Figures 6F,G).

Similar trends were also found in MxCs treated in the same way as ORSCs. As shown in Supplementary Figure S3A, we also observed that DP-EVs transferred miR-140-5p into MxCs. MxCs treated with miR-140-5p–overexpressing EVs had the highest Ki67 + cell ratio at 48 h (Supplementary Figure S3B; ^∗∗^*p* < 0.001). These results indicated that vesicular miR-140-5p stimulates ORSC proliferation. Similar western blot results indicated that ORSCs treated with miR-140-5p–overexpressing EVs expressed the lowest levels of BMP2 and p-SMAD5, and the highest levels of Cyclin D1 and Cyclin E at day 5 (Supplementary Figures S4A,B). Together, these data suggest that DP-EVs promote ORSCs and MxCs proliferation by delivering miR-140-5p, which targets BMP2 and further regulates phosphorylation of SMAD5. Prior studies have confirmed the strong relationship between BMP inhibition and epithelial cell proliferation (Kulessa et al., 2000). These results indicated that vesicular miR-140-5p stimulates ORSC proliferation by inhibiting the BMP signaling pathway.

### DPC-Derived Vesicular miR-140-5p Promotes Hair Growth in Culture

**FIGURE 7:**
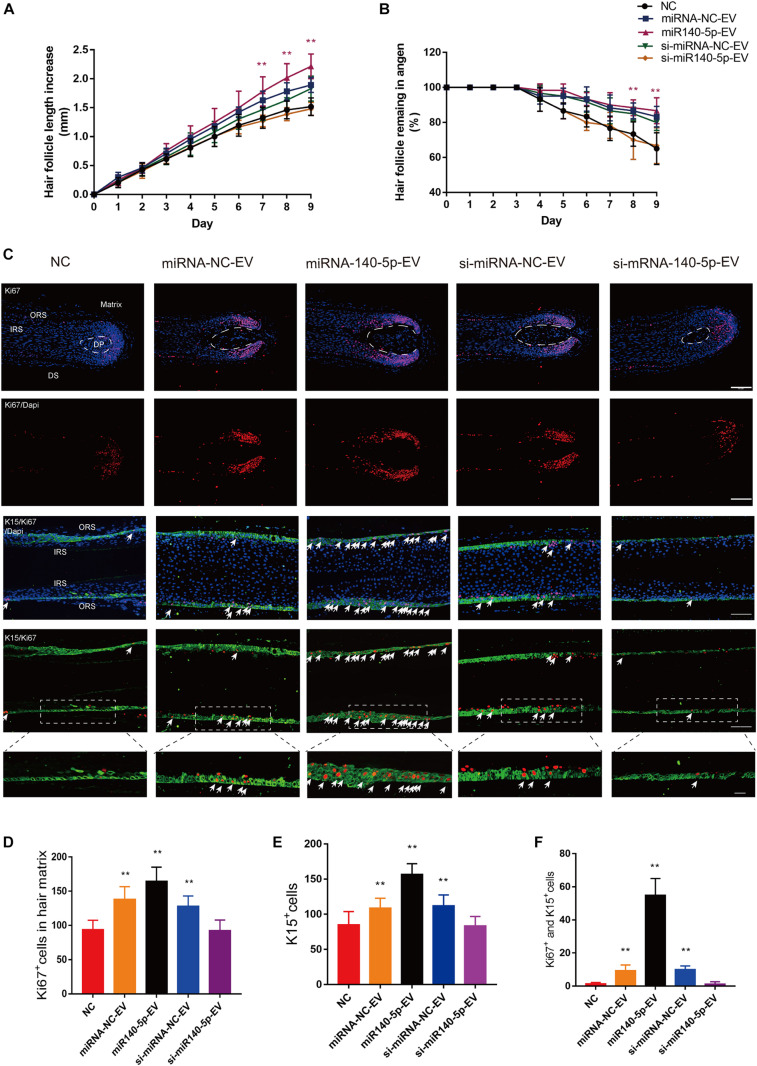
Effect of DPC vesicular miR-140-5p on cultured hair follicles. Cultured human follicles were treated with EVs isolated from DPCs overexpressing (miR-140-5p) or inhibiting miR-140-5p (si-miR-140-5p) for 9 days. The control group was transfected with an equal volume of control Lentiviral vector (miR-NC, si-miR-NC) or PBS. **(A,B)** Hair follicles in each group were assessed and measured daily for increase in hair length **(A)** and the percentage of hair follicles remaining in anagen **(B)**. **(C–F)** Immunofluorescence staining of Ki67 and K15 in hair matrix and lower ORS. Scale bar: 100 μm. Quantitative analysis of the number of Ki67+ **(C,D)**, K15+ **(C,E)** and Ki67+/K15+ double-staining cells **(C,F)** in hair follicle. Data are expressed as means ± s.d. n = 60 from five healthy female individuals (12 samples from each individual). **p < 0.01 vs. PBS-treated group (NC). Statistical significance was evaluated by one-way ANOVA with Bonferroni comparisons; *n* = 5 for each group.

Above, we showed that DPC vesicular miR-140-5p promoted ORSC and MxCs proliferation *in vitro*. However, the HF is a complex mini-organ. Hence, we investigated whether DPC vesicular miR-140-5p was also pro-proliferative in HFs. Specifically, we used a human scalp follicle culture system to investigate the function of vesicular miR-140-5p in HFs. In these experiments, 40 μg EVs (miRNA-NC-EVs, miR-140-5p-EVs, si-miRNA-NC-EVs, or si-miR-140-5p-EV) was added to the medium of cultured HFs every 3 days. After incubation for 9 days, we found that the administration of miR-140-5p-EV significantly promoted HF growth and prolonged anagen (Supplementary Figure S5 and Figures 7A,B; ^∗∗^*p* < 0.01). The HF length in the miR-140-5p-EV group increased 2.22 ± 0.21 mm, with about 86.67% of HFs remaining in anagen during EV treatment. By contrast, when miR-140-5p was silenced in EVs (si-miR-140-5p-EV), the growth of the HFs and the anagen-prolonging effect were repressed (Figures 7A,B; ^∗^*p* < 0.05).

Cell proliferation in cultured HFs was evaluated at day 5 by IHC. We performed double staining for K15 (green) and Ki67 (red) to identify proliferative ORSCs and MxCs. Figures 7C–F shows that miR-140-5p-EVs significantly promoted lower ORSC proliferation and widened the ORS region relative to the control and miR-140-5p–silenced group. In hair matrix, HFs treated with miR-140-5p-EV contained large numbers of Ki67^+^ cells (^∗∗^*p* < 0.001). These data indicated that vesicular miR-140-5p stimulated lower ORSC and MxC proliferation in cultured human HFs.

**FIGURE 8:**
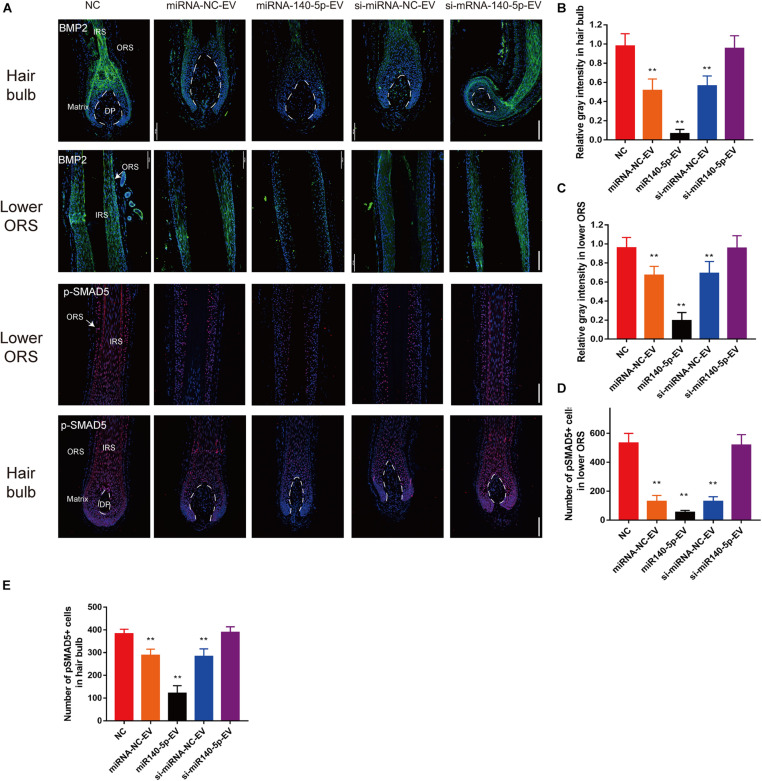
DPC vesicular miR-140-5p downregulated BMP2 and p-SMAD5 in hair follicles. **(A)** Immunofluorescence staining of BMP2 and p-SMAD5 in hair follicle after treatment with DP-EVs for 9 days. Scale bar: 100 μm. **(B–D)** Quantitative analysis of the relative gray intensity level of BMP2 in hair bulb **(B)** and in lower ORS **(C)**, and the number of p-SMAD5^+^ cells in lower ORS **(D)** and hair bulb **(E)**. Data are expressed as means ± SD. *n* = 60 from five healthy female individuals (12 samples from each individual). **p < 0.01 vs. PBS-treated group (NC). Statistical significance was evaluated by one-way ANOVA with Bonferroni comparisons; *n* = 5 for each group.

To confirm the downstream target of miR-140-5p in cultured HFs, we performed IF to analyze BMP2 and p-SMAD5 on day 5. Relative to si-miR-140-5p-EV treatment and the control, BMP2 and its downstream target p-SMAD5 expression decreased in response to miR-140-5p-EV treatment in both the hair matrix and lower ORS region, as demonstrated by immunofluorescence staining (Figures 8A–E; ^∗∗^*p* < 0.01). However, silencing the expression of miR-140-5p in DP-EVs restored the expression of BMP2 and p-SMAD5 (Figures 8A–E; ^∗∗^*p* < 0.01). These data indicated that BMP2 and SMAD5 levels were significantly downregulated in cultured HFs treated with miR-140-5p–overexpressing DP-EVs.

### DPC-Derived Vesicular miR-140-5p Accelerate the Telogen-to-Anagen Transition in C57BL/6 Mice

We also investigated whether DPC vesicular miR-140-5p was pro-proliferative *in vivo*. For this purpose, we used C57BL/6 mice for treatment by depilating a patch of dorsal hair. After treatment with EVs (miRNA-NC-EVs, miR-140-5p-EVs, si-miRNA-NC-EVs, or si-miR-140-5p-EV) (4 mg/ml dissolved in PBS, 1 ml per mouse) or PBS (as a negative control) (Supplementary Figure S6A), the hair coverage rate at day 18 was ∼84.6% in the miR-140-5p-EV-treated group, which was about 1.77-fold higher than PBS-treated group. The results revealed that miR-140-5p-EVs were the most effective at accelerating the hair transition from the telogen to anagen phase (Supplementary Figures S6B,D; ^**, ##,++^*p* < 0.01). The skin thickness of miR-140-5p-EV-treated mice significantly increased relative to the PBS-treated group at day 12 (Supplementary Figures S6C,E). Moreover, miR-140-5p-EV treatment significantly increased the number of Ki67-positive cells (*P* < 0.01) on day 18, whereas the BMP2 level significantly decreased (Supplementary Figures S6F–H). These data revealed that miR-140-5p-EVs were the most effective at accelerating the hair transition from the telogen to anagen phase *in vivo*.

## Discussion

The HF, a mammalian mini-organ, has an intrinsic stem cell niche that regulates prominent physiological growth cycling, making it a valuable model for studying cellular and signaling mechanisms underlying organ regeneration (Wang et al., 2015), as well as disorders of stem cells and their niche, as abnormal secretion from these cells results in hair loss (Ojeh et al., 2015; Pratt et al., 2017; Katzer et al., 2019). Although this issue affects millions of people, the available means of treatment remain limited (Katzer et al., 2019).

The DP are considered to be the signaling center of the HF, which plays an important role in hair growth, cycling, and regeneration (Ojeh et al., 2015; Owczarczyk-Saczonek et al., 2018). It is well established that DPCs induce HF morphogenesis (Biernaskie et al., 2009; Ojeh et al., 2015; Owczarczyk-Saczonek et al., 2018): when mixed with infantile keratinocytes in a specific proportion, implanted DPCs can induce the growth of new HFs in rodents (Amici et al., 2009), and experiments in immune-deficient rats have shown that cells derived from the DP can be incorporated into existing DPs, giving rise to stronger HFs (Biernaskie et al., 2009). However, the HF induction capacity of DPCs decreases with passage (Zhang et al., 2014; Lin et al., 2016; Wang J. et al., 2018); the limited availability of donor hair, ethical concerns, and the risks of immunological rejection also restrict the clinical application of this approach (Zhu et al., 2017; Liu et al., 2018). Recent research has shown that paracrine signaling strongly contributes to the efficacy of cell-based therapies, and that changes in the biological information (such as proteins and miRNAs) carried by EVs may play a critical role in this phenomenon (Witwer et al., 2013). Although DP-EVs from low-passage DPCs can promote the catagen-to-anagen transition of HF and prolong anagen (Chen et al., 2020), we found that DP-EVs from high-passage DPCs lose their ability promote hair growth. The cause for this discrepancy remains unclear, and the underlying mechanism needs to be further investigated.

Hair follicle growth relies on a dermal–epidermal interaction in the HFs (Wang et al., 2015). In anagen, DPCs secrete various activators to the HF epithelium (Yang and Cotsarelis, 2010). During this period, hair growth activation primarily relies on the proliferative activity of the hair matrix and hair ORS, which are located adjacent to DPs (Lee and Tumbar, 2012; Premanand and Reena, 2018). We previously reported that after treatment with DP-EVs, MxCs undergo rapid proliferation and then differentiate to form the hair fiber and IRS (Chen et al., 2020). However, it has not been demonstrated that hair matrix can affect the growth of ORS, and the reason for the accelerated growth of ORS has not been clarified. Several cellular pathways, including BMP/TGF-β, are essential for regulation of epithelial proliferation and differentiation (Kulessa et al., 2000; Lee and Tumbar, 2012; Huang et al., 2019). In the canonical SMAD-dependent BMP signaling pathway, the interactions between BMP2 and its receptors cause phosphorylation of their immediate downstream targets (SMAD proteins); BMP2 was originally defined by its ability to induce ectopic bone and cartilage formation *in vivo* (Reddi, 1992). In the hair formation process, BMP pathway inhibition is a distinguishing feature of the activation of proliferation by epithelial components (Greco et al., 2009; Genander et al., 2014) and can promote WNT signaling (Jamora et al., 2003). Hence, we then focused on whether biological functions differed between EVs derived from high- and low-passage DPCs. We successfully isolated DP-EVs, labeled EVs, and removed excess dye to an undetectable level as previously described (Roberts-Dalton et al., 2017; Krause et al., 2018), and then observed the uptake of DP-EVs in the lower ORS and hair matrix in cultured HFs (Chen et al., 2020). Cell proliferation was stimulated in both cell populations by low-passage human DP-EVs *in vivo* and *in vitro*, but remained unchanged after treatment with high-passage DP-EVs. Hair matrix proliferation is intimately related to the regulatory function of DP, and determines hair shaft and IRS growth (Lee and Tumbar, 2012; Huang et al., 2019). Apart from MxCs, immunofluorescence staining and RT-PCR revealed that lower ORSCs were also dynamically activated after treatment with P3-DP-EVs, which explained the accelerated growth of the hair outer root sheath. We then examined known follicle-regulatory proteins in the process of EV treatment and found that Wnt signaling was upregulated, whereas BMP2 was downregulated, by P3-DP-EV treatment. One possible reason for this difference is that DPC-derived EVs can carry pro-proliferative and hair-inductive information from their parent cells, but this information is gradually lost over the course of serial passage (Biernaskie et al., 2009; Higgins et al., 2013).

The mRNA, miRNA, and protein composition of EVs differ from those of their originating cells. Accumulating evidence shows that miRNAs in EVs play major roles in cell-to-cell communication (Yu et al., 2016). Not only DP-specific markers, but also the whole transcriptome characteristics of DPCs change rapidly over the course of cell passage (Higgins et al., 2013), which may subsequently change the composition of DP-EVs. Thus, to identify the stimulatory factors responsible for EV-regulated HF regeneration that may change over the course of DP cell passage, we performed miRNA sequencing in DP-EVs from low- and high-passage DPCs. Analysis of the 100 most DEMs revealed that the predicted target genes were enriched in functions related to the BMP signaling pathway, and the most significant interaction module identified from among the predicted target genes consisted of BMP signaling components. BMP2 was identified as the hub regulatory gene of miR-140-5p, which accounted for 6.96% of the total miRNA reads in P3-DP-EVs. Although few studies have investigated the role of miR-140-5p in dermatology, miR-140-5p expression is known to promote proliferation in dental pulp stem cells (Sun et al., 2017), neural stem cells (Tseng et al., 2019), and chondrocytes (Tao et al., 2017; Wang Z. et al., 2018). We showed that overexpression of miR-140-5p in human HFs was confirmed to stimulate ORSC and MxCs proliferation by treatment with overexpressed vesicular miR-140-5p, but was restricted by vesicular miR-140-5p inhibition. We also got similar results in animal experiments.

**SCHEME 1:**
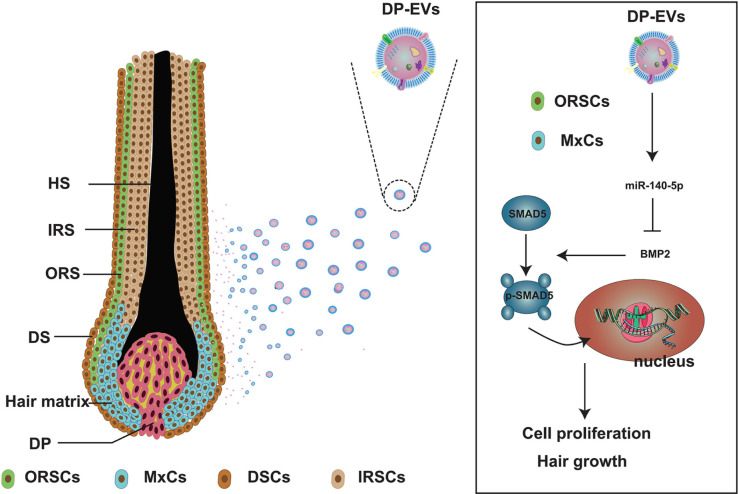
Schematic illustration of low-passage DP-EVs inhibiting the BMP signaling pathway in ORSCs and MxCs. In cultured scalp follicles, miR-140-5p–enriched EVs derived from DPCs were transferred to ORSCs and MxCs. miR-140-5p could suppress the BMP pathway by directly binding to and repressing BMP2. Down-regulation of endogenous BMP2 resulted in inhibition of SMAD5 serine phosphorylation in ORSCs and MxCs, leading in turn to cell proliferation and hair growth. ORSC, outer root sheath cell; MxC, hair matrix cell; DSCs, dermal sheath cell; IRSC, inner root sheath cell; DPC, dermal papilla cell; DP-EV, dermal papilla-derived extracellular vesicle.

BMP2 is a BMP signal that is crucial for maintaining the hair cycle during telogen (Kobielak et al., 2007). Upon BMP inhibition, the HF tilts toward anagen (Greco et al., 2009; Kandyba et al., 2013). In the beginning of anagen, accumulated BMP-inhibitory and Wnt-/Shh-activating signals in HFs initiate a new round of hair growth (Jamora et al., 2003; Shimomura and Christiano, 2010; Hsu et al., 2014). The BMP pathway in HFs is largely antagonistic to the Wnt and Shh pathways: Wnt and Shh signaling are putative BMP targets in epithelial components. Reduced BMP signaling in ORS and hair matrix could lead to proliferation of ORSCs and MxCs (Kulessa et al., 2000; Kobielak et al., 2003), which may help to explain why both ORSC and MxCs proliferation is activated following DP-EV treatment. However, when BMP signaling was downregulated in cultured HFs, changes in the proportion of Wnt/BMP prolong proliferation and could delay differentiation (Plikus et al., 2008; Kandyba et al., 2013). As a result, the anagen stage is prolonged (Plikus et al., 2008). In this study, we demonstrated that BMP2 is a direct target of miR-140-5p, and that overexpression of miR-140-5p in ORSCs and MxCs downregulates BMP2. We then identified and validated bona fide downstream targets of SMAD5, whose phosphorylation is impacted by BMP signaling. Interestingly, gene expression of SMAD5 remained unchanged, but the levels of the phosphorylated product p-SMAD5 were elevated. These data suggested that miR-140-5p directly binds to and regulates BMP2 mRNA but not SMAD5 mRNA. Activation of BMP2 leads to phosphorylation of SMAD5, which ultimately promotes ORSC and MxCs proliferation and prolongs anagen (Scheme 1).

Recent research has discovered that dermal exosomes containing miR-218-5p could promote hair regeneration by regulating β-catenin signaling in mice (Hu et al., 2020). Consistent with our study, miR-140-5p was upregulated 1.64 ± 0.684 fold in 3D cultured DPCs compared with 2D cultured high-passage DPCs based on the previously published sequencing results (Hu et al., 2020). While there was no significant difference in miR-218-5p content in our sequencing results, which may because of the species differences in human and mice HFs, and/or because the miR-218-5p content in DP-EVs is mainly affected by DPC culture environment (3D or 2D) rather than the number of cell passages. It was previously reported that miR-218-5p directly targets SFRP2, and thus, it up-regulates the Wnt/β-catenin pathway in HF in mice (Zhao et al., 2019; Hu et al., 2020). Wnt signaling can also be upregulated in the epithelial population following BMP inhibition (Kulessa et al., 2000; Kandyba et al., 2013). This may help to explain why Wnt signaling in ORSCs and MxCs were also upregulated after treatment with low-passage DP-EVs in our study.

Although the mechanism underlying the therapeutic effects of DP-EVs has not been clarified, our data revealed that low-passage DP-EVs promote ORSC and MxCs proliferation and prolong anagen via vesicular miR-140-5p–mediated suppression of BMP2 signaling pathways. Further investigation is needed to determine whether vesicular proteins and miRNAs other than miR-140-5p impact HF growth and hair cycling. Overall, our findings suggested that DP-EVs could be further exploited as a therapeutic agent to treat hair loss.

## Data Availability Statement

The datasets presented in this study can be found in online repositories. The names of the repository/repositories and accession number(s) can be found in the article/Supplementary Material.

## Ethics Statement

The studies involving human participants were reviewed and approved by Medical Ethical Committee of Southern Medical University. The patients/participants provided their written informed consent to participate in this study. The animal study was reviewed and approved by Experimental Animal Centre at Southern Medical University. Written informed consent was obtained from the individual(s) for the publication of any potentially identifiable images or data included in this article.

## Author Contributions

All authors contributed to writing the manuscript and given approval to the final version of the manuscript. YC, JH, and ZL were principal investigator of the study, conceptualized the research, collected the data, performed the data analysis, and drafted the manuscript. LY, DF, RC, LD, JW, and LW helped to conceive the study and assisted in development of data analysis. YM and ZH provided the supervision and suggestions.

## Conflict of Interest

The authors declare that the research was conducted in the absence of any commercial or financial relationships that could be construed as a potential conflict of interest.

## Footnotes

1 www.omicshare.com/tools

2 http://www.targetscan.org/

3 http://mirdb.org/miRDB/

4 https://string-db.org/

5 https://www.ncbi.nlm.nih.gov/geo/query/acc.cgi?acc=GSE136241

## Abbreviations

EVs: extracellular vesicles
HF: hair follicle
DP: dermal papilla
DPC: dermal papilla cell
DP-EV: dermal papilla-derived extracellular vesicle
ORS: outer root sheath
MxC: hair matrix cell
IRS: inner root sheath
hDPC: human dermal papilla cell
miRNA: microRNA
MSC: mesenchymal stem cell
ALP: alkaline phosphatase
NCAM: neural cell adhesion molecule
α-SMA: α-smooth muscle actin
IF: immunofluorescence
NTA: nanoparticle tracking analysis
P3: passage 3
P8: passage 8
TGF-β: transforming growth factor-beta
WNT: wingless-type MMTV integration site family
BMP: bone morphogenetic protein
K15: keratoprotein 15
3D: three-dimensional.
