# Supplementary material for: miR-140-5p in Small Extracellular Vesicles From Human Papilla Cells Stimulates Hair Growth by Promoting Proliferation of Outer Root Sheath and Hair Matrix Cells
Source: Front Cell Dev Biol. 2020 Dec 14;8:593638. doi: 10.3389/fcell.2020.593638 (PMC7793747; doi:10.3389/fcell.2020.593638)
Supplement: Supplementary file 8 [file Data_Sheet_1.PDF]

Supporting Information

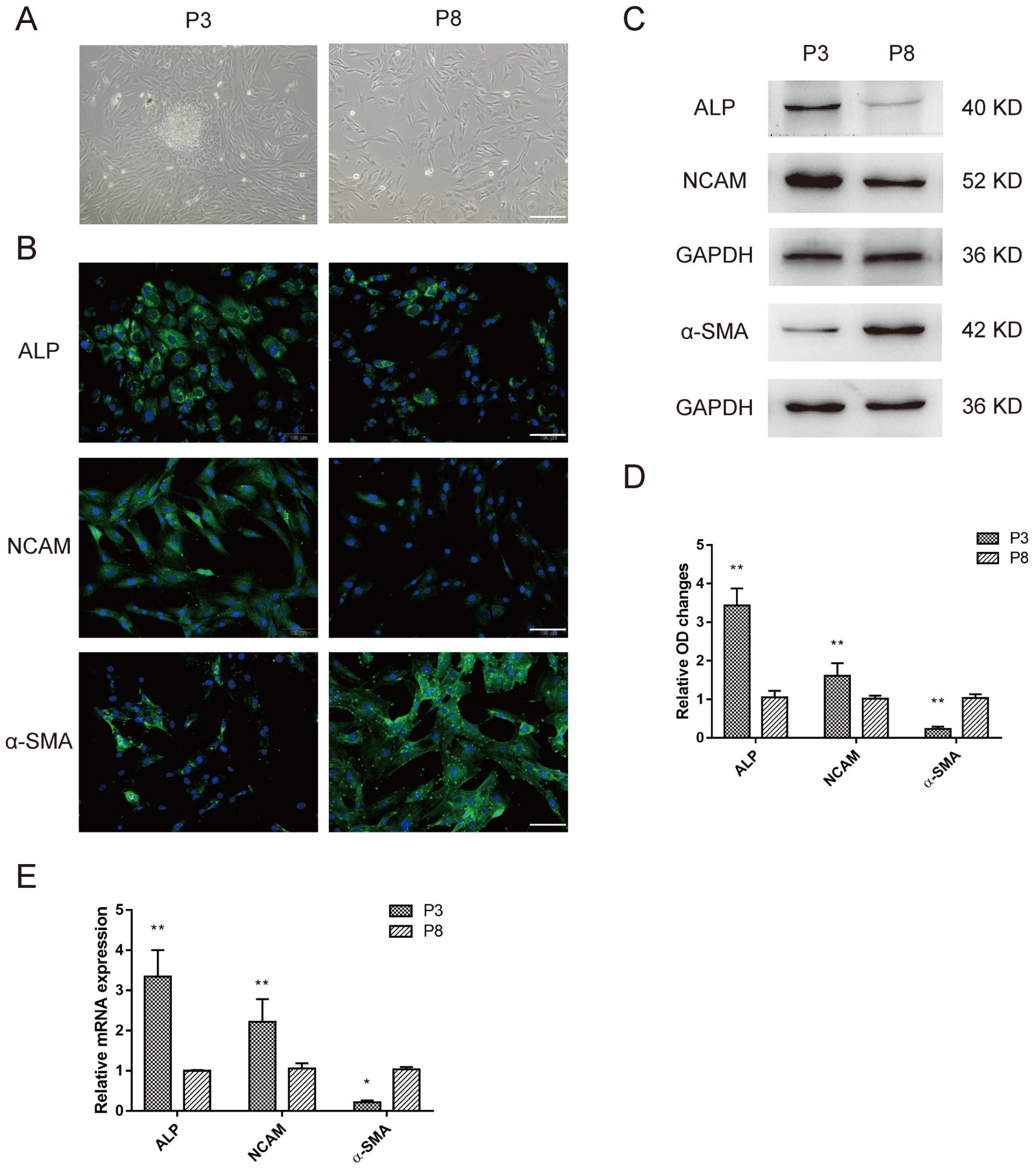

Figure S1. Difference between low- and high-passage DPCs. (A) Morphology of P3 and P8 dermal papilla cells (DPCs) under light microscopy. **(B)** Fluorescence microscope morphology of DPCs in P3 and P8, revealed by staining of DPC-specific markers ALP, NCAM, and α-SMA. Scale bar: 40 μm. **(C,D)** DPC-specific antigens ALP, NCAM, and α-SMA, measured by western blot (**c**) and quantified based on band OD **(D)**. **(E)** Levels of ALP, NCAM, and α-SMA in P3- and P8- DPCs were analyzed by qRT-PCR. Data are expressed as means ± s.d. n = 9, *p < 0.05 vs. PBS-treated group; **p < 0.01 vs. P8-DPCs. Statistical significance was evaluated by one-way ANOVA with Bonferroni comparisons; n = 8 for each group.

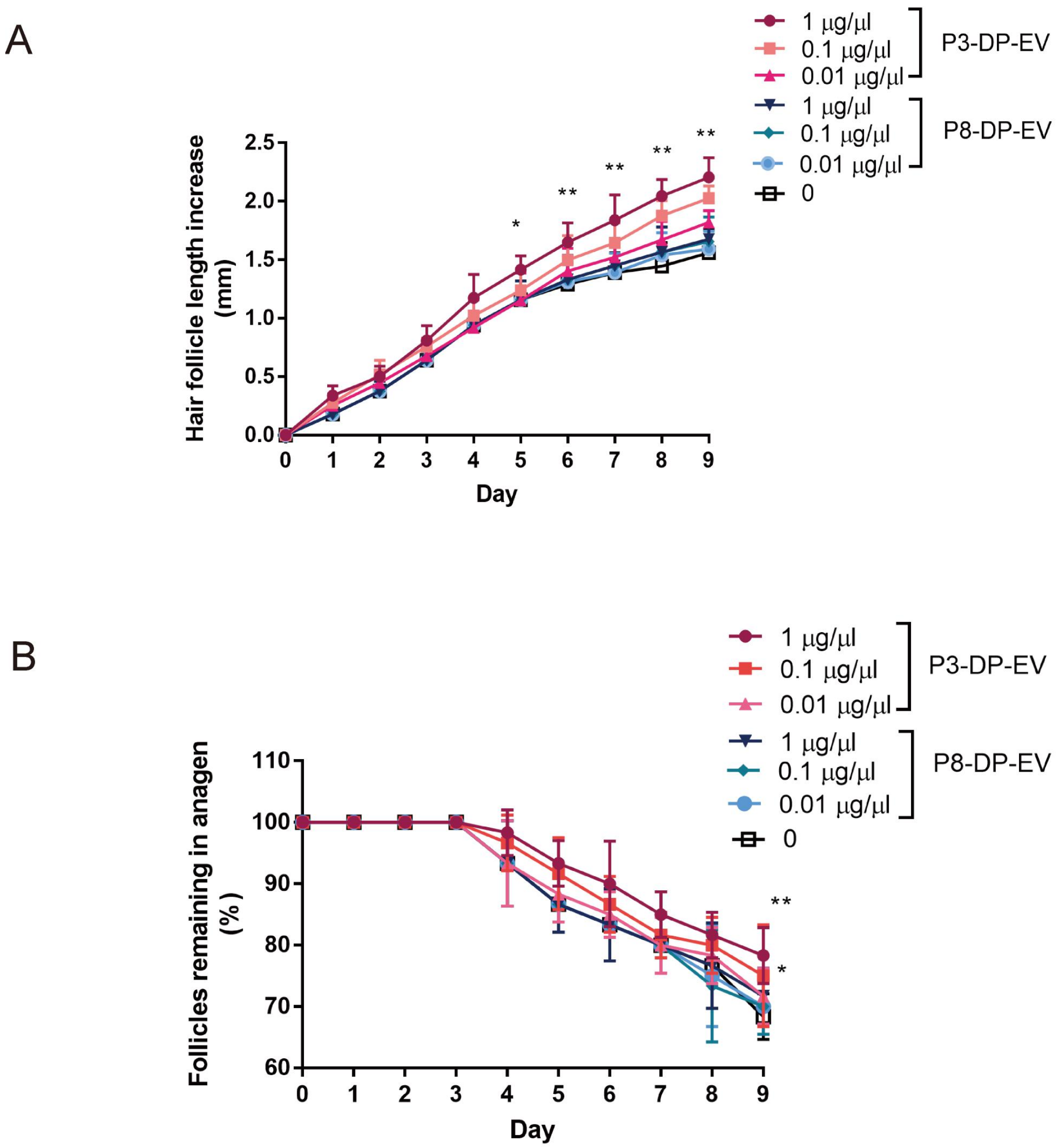

Figure S2. DP-EVs stimulate scalp hair follicle growth in organ culture in a concentration-dependent manner. (A) Hair follicles were treated with 1, 0.1, or 0.01 μg/μl P3- or P8-DP-EVs. At a concentration of 1 μg/μl, P3-DP-EVs significantly increased hair follicle growth ($p < 0.01$) **(B)** Percentage of follicles remaining in anagen upon treatment with the indicated concentrations of P3- or P8- DP-EVs. Data are expressed as means ± s.d. Five individuals were used in each experiment, and 12 follicles/person were examined for each condition. *$p < 0.05$ vs. control; **$p < 0.01$. Statistical significance was evaluated by ANOVA with Bonferroni correction.

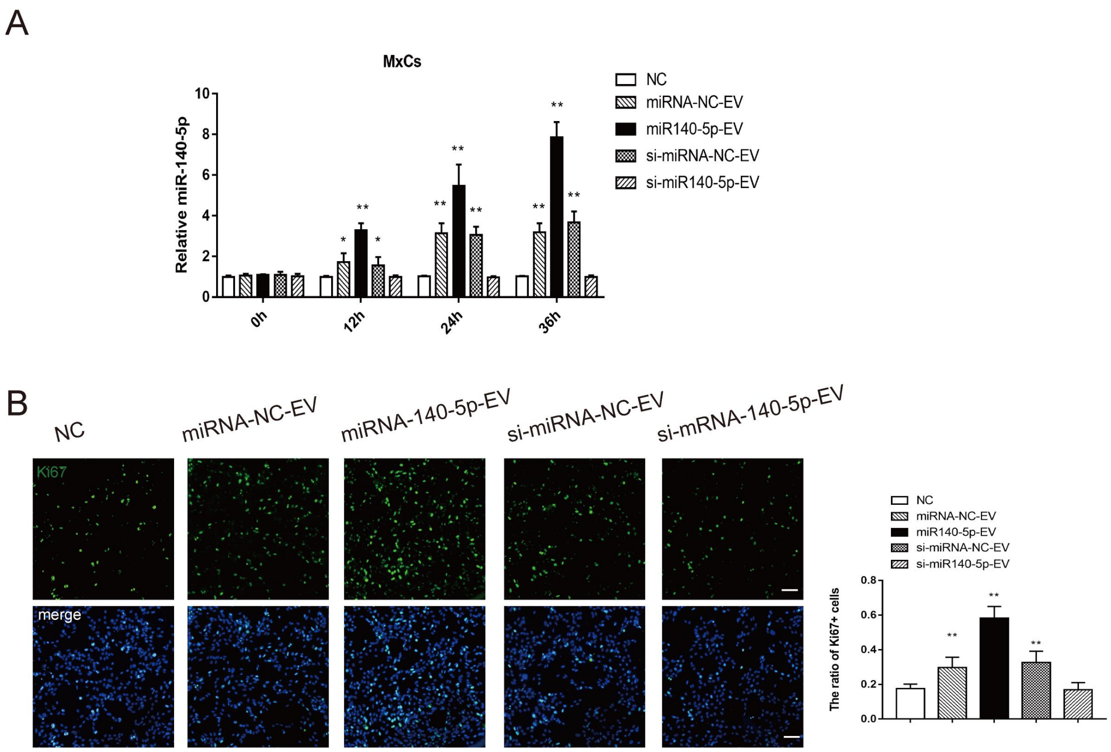

Figure S3. Effect of DPC vesicular miR-140-5p on MxCs *in vitro*. DP-EVs were isolated from DPCs over-expressing (miR-140-5p) or inhibiting miR-140-5p (si-miR-140-5p) for 48 h (concentration for miRNA mimics or inhibitor, 50 nM). The control group was transfected with an equal volume of control lentiviral vector (miR-NC, si-miR-NC) or PBS. **(A)** RT-PCR was performed to analyze the relative expression of miR-140-5p in DPCs and their EVs. **(B)** MxCs were treated with DP-EVs for 48 h. Immunofluorescence micrographs and quantitative analysis of the percentage of Ki67+ MxCs are shown. Scale bar: 50 μm. Data represent means ± s.d. of triplicates (*p < 0.05; **p < 0.01).

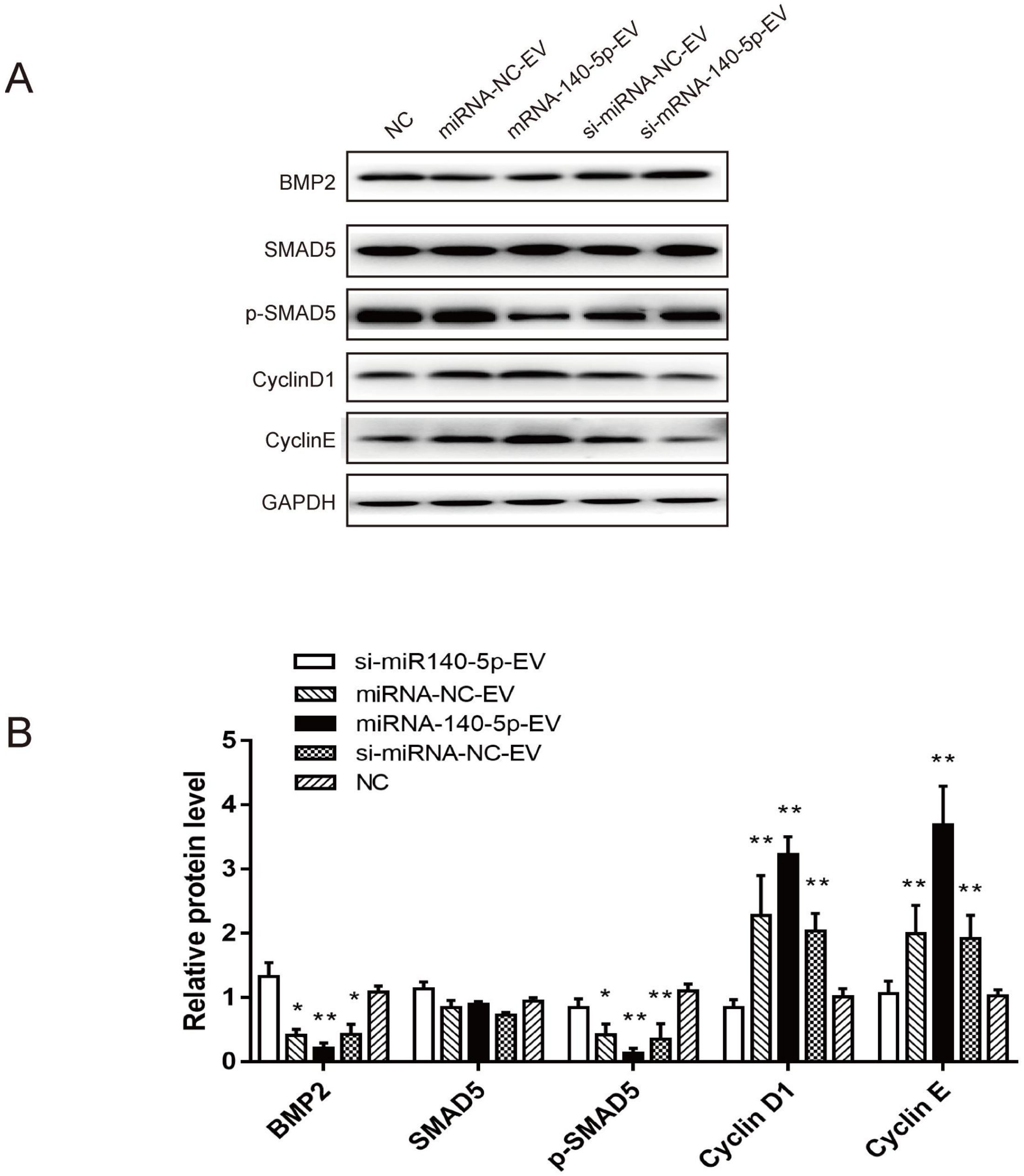

Figure S4. Effect of DPC vesicular miR-140-5p on BMP pathway and cell proliferation. (A,B) Western blot and quantification of BMP-regulatory proteins and cyclins in MxCs after DP-EV treatment for 5 days. Data represent means ± s.d. of triplicates (*p < 0.05; **p < 0.01).

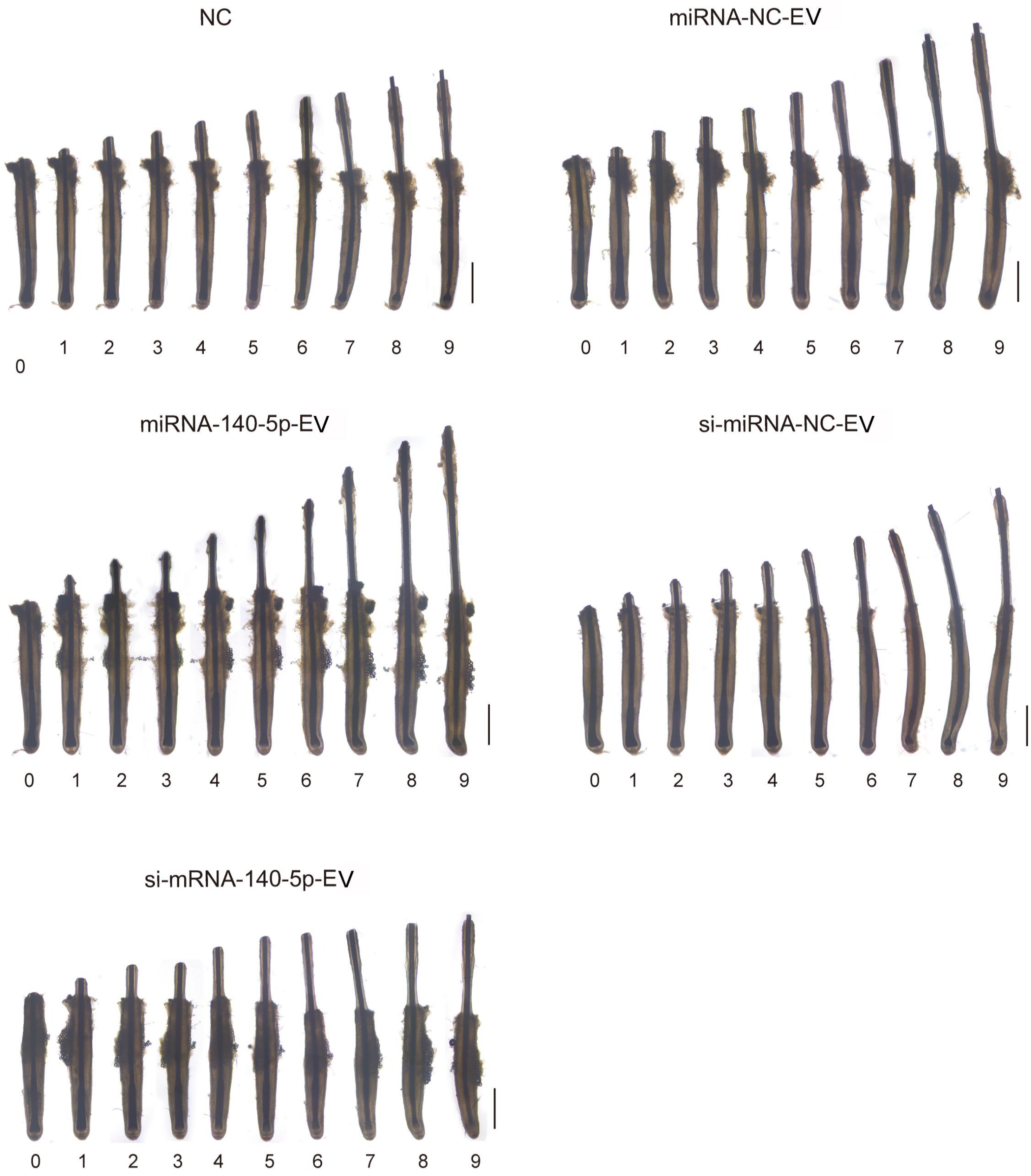

Figure S5. Sequential photomicrographs of human scalp hair follicles growing in organ culture. Follicles were cultured in control medium (NC), 1 μg/μl miR-NC-EVs, 1 μg/μl miR-140-5p-EVs, 1 μg/μl si-miR-NC-EVs, or 1 μg/μl si-miR-140-5p-EVs. Sequential photomicrographs of individual scalp follicles in organ culture under various conditions were acquired every 24 h for 9 days. Scale bars: 1 mm.

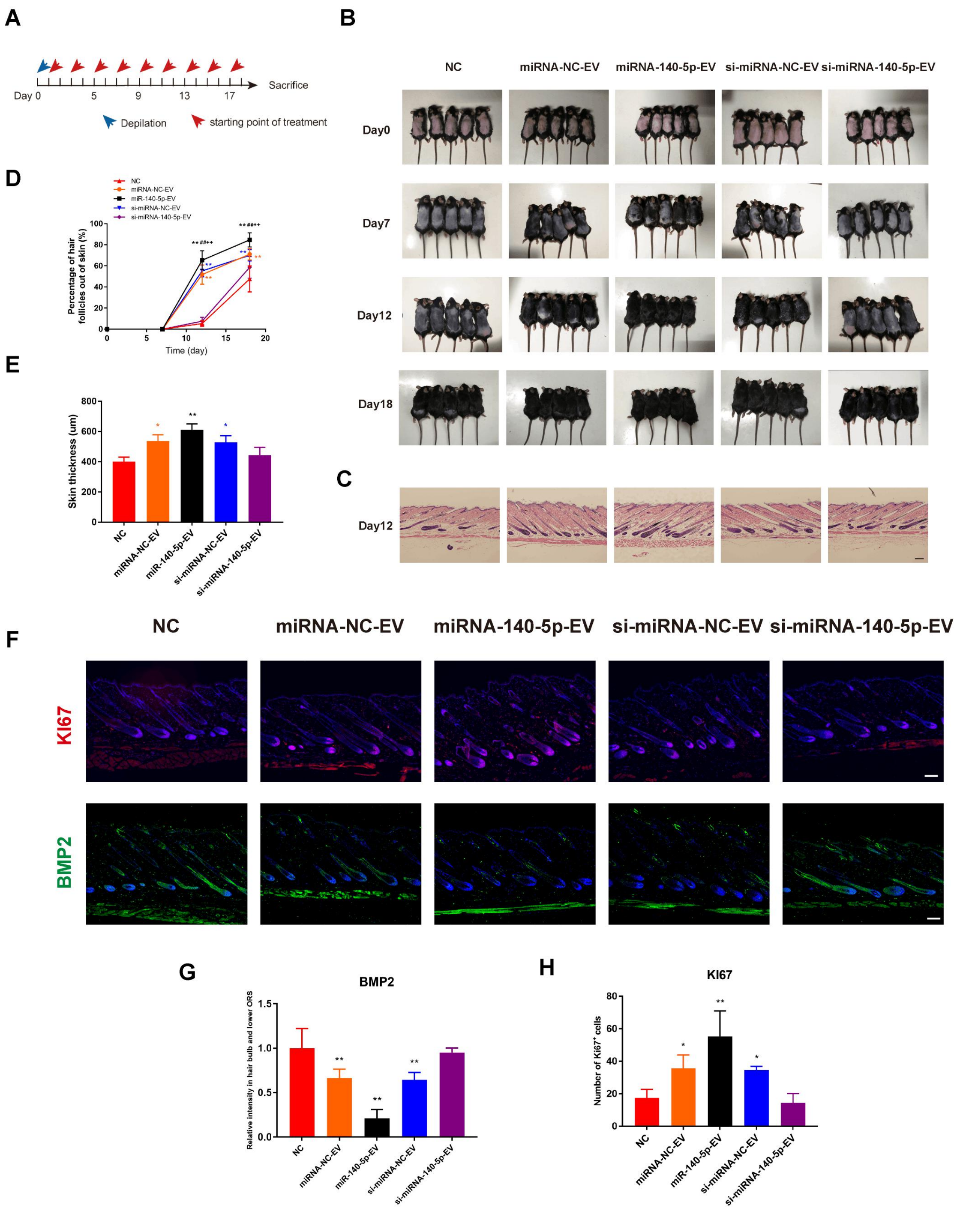

Figure S6. DPC vesicular miR-140-5p accelerate the telogen-to-anagen transition in C57BL/6 mice. (A) After hair depilation, mice dorsal skin was treated every 4 days with EVs (miRNA-NC-EVs, miR-140-5p-EVs, si-miRNA-NC-EVs, or si-miR-140-5p-EV). Observation continued over an 18-day treatment period, then animals were sacrificed for further analysis. (B) The dorsal skin was photographed at 0, 7, 12, and 18 days. (C) HE-stained sections of dorsal skin at 12 days. Scale bars: 50 μm. (D) Areas with hair regrowth out of skin were quantified using Image-Pro Plus software. (E) miR-140-5p-EV-treated mice were advanced, with a significant increase in skin thickness relative to the PBS-treated groups at day 12. (F) Immunofluorescence staining of skin at day 18. Nuclei are stained with DAPI (blue). Scale bars: 50 μm. (G-H) BMP2

and Ki67 expression on day 18 was highest in the miR-140-5p-EV-treated group. Each bar represents the mean ± S.D of eight replicates. *, $p < 0.05$ relative to the negative control; **, $p < 0.01$ relative to the negative control; ##, $p < 0.01$ relative to miRNA-NC-EV group; ++, $p < 0.01$ relative to si-miRNA-NC-EV group. Statistical significance was determined by one-way ANOVA with Bonferroni correction; n = 8 for each group.

Table S1. Primers used for real-time PCR analysis

**Genes**	**Forward (5′–3′)**	**Reverse (5′–3′)**
β-catenin	CTGAGGAGCAGCTTCAGTCC	ATTGCACGTGTGGCAAGTTC
Lef1	GCTCCTGAAATCCCCACCTTC	GGATGAGGGATGCCAGTTGTG
Tcf3	GCACTGGCCTCGATCTACTC	TTCAGGATGACCGAGACAGC
SHH	TCGCAGCTGCTCTACCAAAT	CCGGGGTCCTTGTTTCCTTA
Gli	GACATACCCCACCTCCCTCT	ACTGCAGCTCCCCCAATTTT
PTCH1	GCTACTTACTCATGGTTTTGCCA	TGTCGTGTGTGTCGGTGTAG
BMP2	AGCCCAGTTGCTGCTCCAGGTCC	AAGACAGCGGGTCCCGGCCA
BMPR1A	TGCAGAGATTGGAATCCGCC	GACCTATGACAACAGGGGGC
SMAD1	TGCCTCACGTCATCTACTGC	ATGCACACCTCCTTCTGCTT
SMAD5	TAGCCGGCTCGCGAAAAG	GGGTCAAGTCAGAGGCAGATTT
SMAD8	CTGGAAGCAGGGAGATGAAG	TGGTGACACACTTGCTAGGC
K15	AGGGCCTGAATGAGGAGCTA	GAAGAGGCTTCCCTGATGGC
K19	TGGAGATGCAGATCGAAGGC	GCCCCTCAGCGTACTGATTT
CD200	AGGATGGAGAGGCTGGTGAT	CCGGTGACGTTTCCAGTACA
miR-28-3p	CGCGCACTAGATTGTGAGCT	AGTGCAGGGTCCGAGGTATT
miR-31-5p	CGGCGGAGGCAAGATGCTGGCA	CAACTGGTGTCGTGGAGTCGG
miR-342-3p	TCCTCGCTCTCACACAGAAATC	TATGGTTGTTCACGACTCCTTCAC
miR-382-5p	ATCCGTGAAGTTGTTCGTGG	TATGGTTGTAGAGGACTCCTTGAC
miR-452-5p	AGCGCGAACTGTTTGCAGAGGA	ATCCAGTGCAGGGTCCGAGG
miR-10b-5p	UACCCUGUAGAACCGAAUUUGUG	AGTGCAGGGTCCGAGGTATT
miR-454-3p	GCGCGTAGTGCAATATTGCTTA	AGTGCAGGGTCCGAGGTATT
miR-140-5p	GCCTCAGTGGTTTTACCC	GTGCAGGGTCCGAGGT
miR-18a-3p	GCTGAGCTAAGGTGCATCTA	TCAACTGGTGTCGTGGAGT
miR-23a-5p	GGGGTTCCTGGGGATGGGATTT	AGTGCAGGGTCCGAGGTATT
U6	CTCGCTTCGGCAGCACA	AACGCTTCACGAATTTGCGT
GAPDH	TGGGGTGAGGCCGGTGCTGA	GGCATCGGCAGAAGGGGCGG
